# Pollination and seed dispersal are the most threatened processes of plant regeneration

**DOI:** 10.1038/srep29839

**Published:** 2016-07-20

**Authors:** Eike Lena Neuschulz, Thomas Mueller, Matthias Schleuning, Katrin Böhning-Gaese

**Affiliations:** 1 Senckenberg Biodiversity and Climate Research Centre Frankfurt, Senckenberganlage 25, 60325 Frankfurt am Main, Germany; 2 Goethe Universität Frankfurt, Fachbereich Biowissenschaften, Max-von-Laue-Str. 9, 60438 Frankfurt am Main, Germany

## Abstract

Plant regeneration is essential for maintaining forest biodiversity and ecosystem functioning, which are globally threatened by human disturbance. Here we present the first integrative meta-analysis on how forest disturbance affects multiple ecological processes of plant regeneration including pollination, seed dispersal, seed predation, recruitment and herbivory. We analysed 408 pairwise comparisons of these processes between near-natural and disturbed forests. Human impacts overall reduced plant regeneration. Importantly, only processes early in the regeneration cycle that often depend on plant-animal interactions, i.e. pollination and seed dispersal, were negatively affected. Later processes, i.e. seed predation, recruitment and herbivory, showed overall no significant response to human disturbance. Conserving pollination and seed dispersal, including the animals that provide these services to plants, should become a priority in forest conservation efforts globally.

Forest ecosystems worldwide have undergone severe losses in biodiversity and ecosystem functioning^1^,^2^. To foster the resilience and ensure the long-term stability of forests, the regeneration of woody plants is critical^3^. Plant regeneration comprises a cycle of life stages from seeds to seedlings and adult plants^4^. Several processes determine the transitions between these stages. Processes that facilitate transitions include pollination, seed dispersal, and recruitment while processes such as seed predation and herbivory are detrimental^4^. To understand which of these processes are most sensitive to human disturbance, we performed a global comparative assessment across regeneration processes. Such a meta-analysis could reveal potential breaking points in the regeneration cycle, which is essential for prioritizing forest conservation efforts.

Meta-studies to date have compiled evidence about negative effects of human disturbance on plant regeneration, but have focused on single processes, in particular on those early in the plant life cycle, such as pollination and seed dispersal^5^,^6^,^7^. Especially, the pollinator crisis and its potential implications for food security^8^ and the consequences of the loss of large seed-dispersing animals on forest structure and global carbon storage^9^ have received great attention in the scientific community and beyond. In contrast, comparable and quantitative analyses of the effects of human disturbance on later stages of the plant life cycle, e.g. seed predation and recruitment, are missing. Including these later stages is indispensable to identify the processes that are most sensitive to human disturbance globally.

Here we present the first comprehensive study analysing effects of human forest disturbance across ecological processes that are essential for the regeneration of forest ecosystems. We identified 145 studies of 247 woody plant species covering 34 countries and all 12 forest and woodland biomes according to the WWF ecoregion classification^10^. These studies provided a total of 408 comparisons of plant regeneration between protected, natural or near-natural forests and forests disturbed by humans (Fig. 1). We focused on those effects of human activities that have been identified as the most important drivers of forest disturbance^11^,^12^. These included land-use changes such as fragmentation, selective logging and conversion to secondary forest habitat, as well as defaunation such as bushmeat hunting, and compared plant regeneration between near-natural and disturbed forests. We calculated Hedge’s *d* as an estimate of the standardized mean difference for each of the 408 comparisons between near-natural and disturbed forests^13^.

## Results

Overall, we found a significant negative effect of human forest disturbance on plant regeneration. The standardized mean difference between disturbed and undisturbed forests was −0.25 (95% confidence interval (CI) of Hedge’s *d*: −0.44 to −0.07, p = 0.008), underpinning that human forest disturbance reduces plant regeneration generally.

To identify the processes that are most vulnerable to forest disturbance, we distinguished the specific processes in the plant regeneration cycle (i.e. pollination, seed dispersal, seed predation, recruitment and herbivory) and additionally accounted for the latitudinal and longitudinal position of each case study and plant life history (approximated by plant seed size). Previous studies have demonstrated that plant seed size is closely correlated to other plant life history traits, such as tree height or wood density^9^,^14^,^15^. Adding these moderators resulted in a more parsimonious model (delta AIC compared to the model without moderators = 42). In this model, pollination (*d* = −1.12, CI: −1.59 to −0.65, p < 0.001) and seed dispersal (*d* = −0.64, CI: −1.00 to −0.28, p < 0.001) were significantly lower in disturbed than in undisturbed forests (Fig. 2). In contrast, seed predation (*d* = 0.27, CI: −0.13 to 0.66, p = 0.18), recruitment (*d* = −0.28, CI: −0.65 to 0.09, p = 0.14) and herbivory (*d* = −0.05, CI: −0.60 to 0.49, p = 0.85) were not significantly related to forest disturbance. The negative effects of forest disturbance on pollination and seed dispersal were more than twice as strong as the effects on seed predation, recruitment and herbivory. We emphasize that the sample size for each process was high (Fig. 2). Effects of forest disturbance on plant regeneration were independent of latitude (*d* = −0.01, CI: −0.00 to 0.02, p = 0.16) and longitude (*d* = −0.00, CI: −0.00 to 0.00, p = 0.69), indicating that plant responses were similarly strong in tropical and temperate ecosystems as well as across longitudes. Seed size had a significant effect on the response of plants to forest disturbance (*d* = −0.45, CI: −0.83 to −0.07, p = 0.021) with large-seeded plants being more affected by disturbance than small-seeded plant species.

## Discussion

Our study shows that pollination and seed dispersal are the most vulnerable ecological processes in the life cycle of plants; only these processes were significantly affected by forest disturbance at a global scale. A fundamental characteristic of these early processes in plant regeneration is that they usually depend on mutualistic plant-animal interactions. More than 87.5% of angiosperms depend on pollination by animals^16^, and over 75% of tree species in tropical^17^ and 30–40% of tree species in temperate systems^18^ depend on seed dispersal by animals. The most important interaction partners of plants for pollination and seed dispersal include insects, such as bees or hoverflies, birds and mammals. These animal groups have experienced severe declines in past decades through land-use changes, overexploitation and defaunation^7^,^8^,^19^ which likely explain the negative effects of forest disturbance on pollination and seed dispersal shown in this study. Pollinator loss has been shown to cause a reduction in plant seed set and offspring fitness^20^, for instance due to a decrease in pollen diversity and an increase in plant inbreeding^21^,^22^. Similarly, the decline of animal seed dispersers results in reduced seed removal, particularly in large-seeded plant species^5^,^23^. Our findings corroborate that large-seeded species are most prone to the impacts of human forest disturbance. Since large-seeded species are typical for late-successional forest habitats^24^,^25^, this effect underlines that these species are likely to be more sensitive to human disturbance than early-successional species.

Our results give evidence that disruptions of mutualistic interactions between plants and their animal pollinators and seed dispersers are the fundamental threat to plant regeneration in forest ecosystems. In contrast, seed predation, recruitment and herbivory were not significantly related to forest disturbance (Fig. 2). Unlike the early regeneration processes that are influenced by mutualistic interactions, these later processes are either largely mediated by antagonistic interactions (in the case of seed predation and herbivory) or by abiotic conditions (in the case of recruitment). Seed predation and herbivory tend to vary strongly in their responses to human disturbance^23^. For example, defaunation can have both increasing^26^,^27^,^28^ as well as decreasing^23^ effects on seed predation. Likewise, disturbance effects at forest edges often increase herbivory^29^, while disturbance through forest fragmentation usually decreases herbivory^30^. Previous studies show that the low vulnerability of antagonistic interactions to human disturbance may be related to a low degree of specialization in antagonistic interactions^31^, density compensation^32^, or high context dependence^33^. Similar to seed predation and herbivory, responses of plant recruitment to forest disturbance also tend to be variable^34^,^35^. One explanation for this pattern could be the increasing importance of abiotic factors later in the plant life cycle^36^. For instance, the recruitment of plants in disturbed forests can be facilitated by high light availability^37^, but can be reduced by water limitation^37^. Hence, abiotic drivers can facilitate the recruitment of light-demanding, drought tolerant species in disturbed forests, potentially leading to changes in plant species composition, taxonomic homogenization and a reduction in plant diversity^38^. However, our meta-analysis did not detect a greater variability of disturbance effects on recruitment than on earlier regeneration processes (see Fig. 2). This suggests that overall the effects of disturbance on recruitment were indeed weaker and not more variable than those on dispersal or pollination.

Typical recruitment studies, such as those in our meta-analysis, examine effects of forest disturbance on recruitment over short time periods. However, to reveal how disruptions of early regeneration stages influence adult plants and plant communities, long-term studies that track cascading effects through the entire regeneration cycle from dispersal and pollination to adult plants are required. Thus far, very few long-term studies have tested such cascading effects^9^. Long-term exclusion experiments and local extinctions^39^,^40^,^41^,^42^,^43^ as well as studies on regional defaunation^44^,^45^,^46^ all show that the loss of pollinators and seed dispersers can indeed reduce populations of single plant species (Supplementary Fig. 1A) and cause significant changes in the structure and composition of plant communities (Supplementary Fig. 1B). These studies provide crucial evidence that disruptions in the early regeneration cycle of plants will ultimately alter plant population dynamics. Nevertheless, we emphasize the need for more long-term and experimental studies investigating cascading effects in the regeneration cycle, including manipulative experiments^43^ and in-depth studies on the more subtle cascading effects of forest disturbance on genetic structure and spatial patterns of seedling recruitment^23^ and survival^36^.

**Figure 1:**
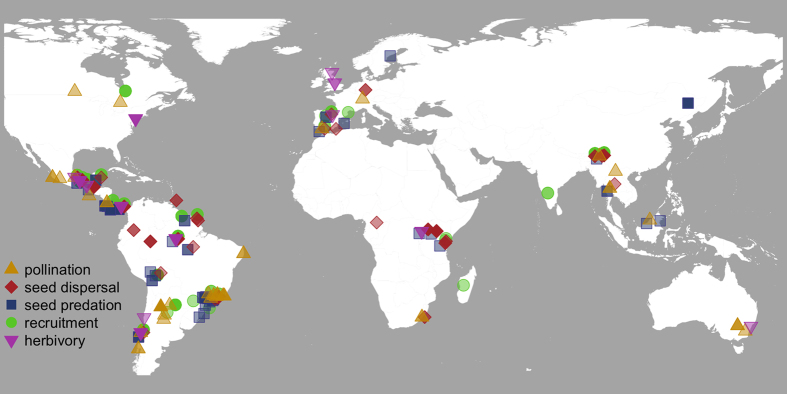
Map of the study sites of all case studies (n = 145) included in the meta-analysis: pollination (n = 32, yellow triangles), seed dispersal (n = 41, red diamonds), seed predation (n = 42, blue squares), recruitment (n = 45, green circles) and herbivory (n = 20, pink triangles). Please note that some study sites cover more than one process of plant regeneration (indicated by darker shading). Map created with the statistical programming language R (Version 3.2.1, https://www.r-project.org)^61^ using the mapdata package^65^.

**Figure 2:**
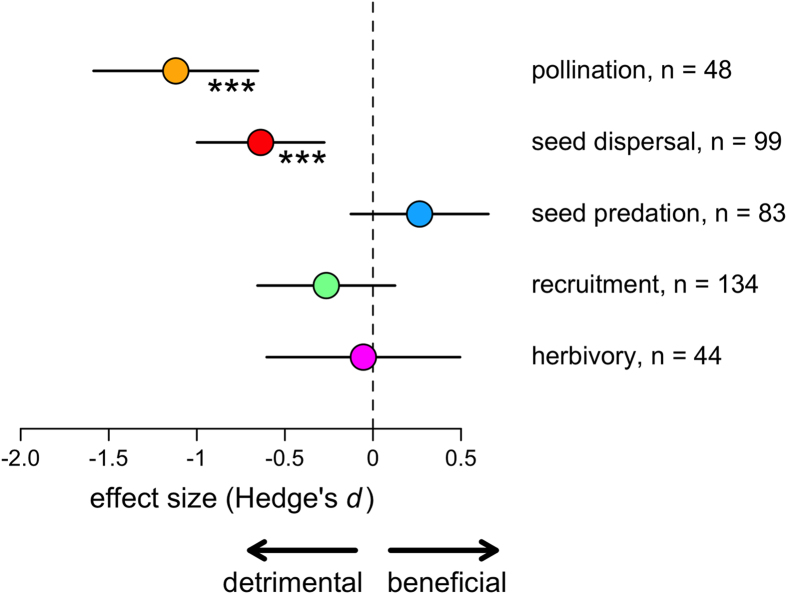
Effects of human forest disturbance on plant regeneration. Shown are mean effect sizes (Hedge’s *d*) and 95% CI for pollination, seed dispersal, seed predation, recruitment and herbivory. Negative effect size indicates a detrimental effect, positive effect size indicates a beneficial effect of forest disturbance on the respective process (***p < 0.001). The model also included absolute latitude, longitude, seed size, and a spatial autocovariate to account for geographic location, plant life history, and spatial autocorrelation, respectively. The model included plant species, genus and study site as random effects. n = number of comparisons per process.

Our meta-analysis demonstrates that pollination and seed dispersal, often mediated by animal species, are overall the most fragile processes in plant regeneration. This pattern was consistent across tropical and temperate ecosystems and across longitudes. However, our study shows that in some regions of the globe the effects of forest disturbance on plant regeneration have been little investigated, such as in temperate and boreal forest ecosystems (Fig. 1). We highlight the need for more studies investigating the effect of human disturbance on plant regeneration processes in these regions. Although we did not restrict our search for publications to particular plant groups, animal-dependent plant species were prevalent in our data set reflecting the dominance of animal-dependent plant species globally^16^,^17^,^18^. We would expect that the relatively small group of plants that neither depend on animals for pollination nor for dispersal, is less affected by forest disturbance than animal-dependent plants, at least in the early steps of plant regeneration. The sensitivity of plant-animal mutualisms lends support to the hypothesis that the loss of animal species in response to forest disturbance is a threat to global biodiversity, due to top-down forcing in ecosystems^47^. Natural forest regeneration and forest restoration activities that accelerate the recovery of forest ecosystems are indispensable to sustain global biodiversity and ecosystem functioning^3^,^48^,^49^. Our study shows that conservation practices should globally focus on the early steps of plant regeneration. As these processes critically depend on mutualistic plant-animal interactions, this implies that forest conservation and restoration efforts must prioritise the protection of animal pollinators and seed disperses.

## Methods

We conducted a systematic search of peer-reviewed journal articles including all records until June 2016 that focussed on the effects of human forest disturbance on ecological processes involved in plant regeneration (i.e., pollination, seed dispersal, seed predation, recruitment and herbivory). We are aware that other processes, such as fungal or soil microbial interactions, may also play a role, but only few case studies have studied their effects on plant regeneration across a disturbance gradient^50^,^51^. At each step of this study we followed the guidelines of quality criteria for meta-analyses listed by Koricheva and Gurevitch^52^. We searched the Web of Science using combinations of the following keywords “((fragmentation OR disturbance OR hunting OR defaunation) AND (pollination OR seed_set OR seed_dispersal OR seed_removal OR seed_predation OR pilferage OR germination OR establishment OR recruitment OR herbivory) AND (tree OR shrub))”. The search resulted in 4,242 journal articles. Furthermore, we added publications that were cited in meta-analyses of the effects of forest disturbance on specific processes (pollination^6^,^53^,^54^; seed dispersal^5^; predation^23^,^55^; recruitment^23^; herbivory^29^,^30^,^56^,^57^).

We searched the abstracts of the pre-selected publications for matching the following criteria: 1) study investigates the effect of forest disturbance on pollination, seed dispersal, post dispersal seed predation, recruitment or herbivory of at least one woody plant species; 2) study compares near-natural or protected forest to one of the following types of disturbed forest: fragmented forest, forest edge, defaunated forest, forests hunted for bushmeat, logged forest, or secondary forest; 3) study reports on observational or experimental field data. We excluded studies in which isolated trees were compared to natural forests, studies in which fire events caused forest disturbance, and studies on cultivated crops or invasive species.

Out of 339 publications that matched these criteria, we retained 145 studies from which we were able to extract mean values, standard deviations and sample sizes of the comparison between near-natural and disturbed forests (Supplementary Dataset). If necessary, we extracted data with graphical software^58^. We chose response variables that have been used in previous meta-analyses and have been proven to reflect the respective processes of plant regeneration. For pollination, we used pollinator visitation and seed set^6^,^54^; for seed dispersal, we used seed-disperser visitation, disperser seed removal and seed-dispersal distance^5^ (we only included primary seed dispersal, since quantitative studies on the effects of human disturbance on secondary seed dispersal are scarce in the literature); for post-dispersal seed predation: rodent seed predation, insect infestation and predator seed removal^23^,^55^ for recruitment: germination, seedling establishment, seedling survival and sapling establishment^23^,^59^ for herbivory: herbivore abundance, leaf damage and leaf loss^23^,^55^.

We used the following protocol for data extraction: (1) In case the original paper studied a gradient of forest disturbances, we used the end-points of the gradient (i.e., continuous forest vs. smallest fragment; forest interior vs. forest edge; protected forest vs. forest with bushmeat hunting; near-natural forest vs. highest degree of logging; near-natural forest vs. secondary forest) to define a two level factor^55^. (2) If several plant species were investigated in the same original study, we included both in the data set and accounted for non-independence with a random effect for study (see data analysis section). Likewise, if the same plant species was investigated in more than one study, we included both in the data set and accounted for non-independence with a random effect for species (see data analysis section). We extracted the latitudinal and longitudinal position of the study site from the original publication and annotated each site with biomes based on the WWF Terrestrial Ecoregions^10^. We also extracted measures of seed size and seed mass from the publications. If no seed measurements were specified in the publication, we either extracted data from other publications on the same plant species, or we obtained seed measurements from the TRY data base (www.try-db.org)^60^. For 92 out of 247 plant species, we could only acquire data on seed mass. We conducted a linear regression using the species for which we had information on both seed size and seed mass (seed size and seed mass both log-transformed, n = 83 species, r^2^ = 0.77, β = 0.32) and predicted the seed size for those species where only seed mass was available (see Supplementary Dataset for predicted seed sizes). We obtained taxonomic classification from The Plant List Version 1.1 (www.theplantlist.org) for all 247 plant species, which belonged to 70 families and 159 genera. Furthermore, we retained information on pollination and seed-dispersal syndromes of each plant species from the publication or additional literature. More than 96% of the plant species in our data set depended on animals for pollination and/or seed dispersal, reflecting the high proportion of animal-dependant plants in natural ecosystems^16^,^17^.

### Data analysis

To examine the effect of forest disturbance on plant regeneration, we built two models that compared disturbed versus non-disturbed forest across all original studies. The first model tested the overall effect of human forest disturbance on plant regeneration. The second model tested whether disturbance differentially affected plant regeneration processes and additionally included absolute latitude and longitude as a measure of the geographic location of the study sites and seed size as a correlate for life-history strategy. We also tested for a potential publication bias that was not detectable (see Supplementary Methods 1).

All analyses were conducted with the statistical programming language R^61^ using the metafor package^62^. We calculated Hedge’s d as an estimate of the mean standardized difference and the corresponding sampling variance for each comparison between near-natural and disturbed forests (see Supplementary Dataset)^62^. This estimator is robust against small sample sizes and unequal sampling variances^13^. In the comparison between near-natural versus disturbed forests, we defined effect sizes as positive when the ecosystem process was beneficial for plant regeneration and negative if it was detrimental for plant regeneration. That is, we characterized more pollination, seed dispersal, recruitment and less seed predation and herbivory with positive effect sizes and vice versa.

We fitted random effects models accounting for variability in sampling methods among studies^62^. Both models included the study site as additional random effect to account for non-independent samples from the same site. We accounted for phylogenetic relatedness by including plant species and genus as random effects. We also added a spatial autocovariate as fixed effect to control for spatial autocorrelation^63^. After including the spatial autocovariate, we could not detect spatial autocorrelation in the model residuals (see Supplementary Methods 2). Effect sizes were weighted by the inverse sampling variances^62^. In both models, we used REML approximation of the model estimates^62^.

To test the robustness of our findings, we additionally built both models using a Bayesian approach^64^. In this case, we accounted for phylogenetic relatedness among plant species with a taxonomic tree and kept the model structure otherwise identical. For both models, the results were qualitatively identical (see Supplementary Methods 3).

## Additional Information

**How to cite this article**: Neuschulz, E. L. *et al*. Pollination and seed dispersal are the most threatened processes of plant regeneration. *Sci. Rep*. **6**, 29839; doi: 10.1038/srep29839 (2016).

## Supplementary Material

Supplementary Information
