# Supplementary material for: Pollination and seed dispersal are the most threatened processes of plant regeneration
Source: Sci Rep. 2016 Jul 20;6:29839. doi: 10.1038/srep29839 (PMC4951728; doi:10.1038/srep29839)
Supplement: Supplementary Information [file srep29839-s1.pdf]

Supplementary Information for **“**Pollination and seed dispersal are the most threatened processes of plant regeneration”

Eike Lena Neuschulz[1*], Thomas Mueller[1,2], Matthias Schleuning[1] and Katrin Böhning-Gaese[1, 2]

[1] Senckenberg Biodiversity and Climate Research Centre Frankfurt, Senckenberganlage 25, 60325 Frankfurt am Main, Germany

[2] Goethe Universität Frankfurt, Fachbereich Biowissenschaften, Max-von-Laue-Str. 9, 60438 Frankfurt am Main, Germany

*corresponding author, contact: elneuschulz@gmail.com

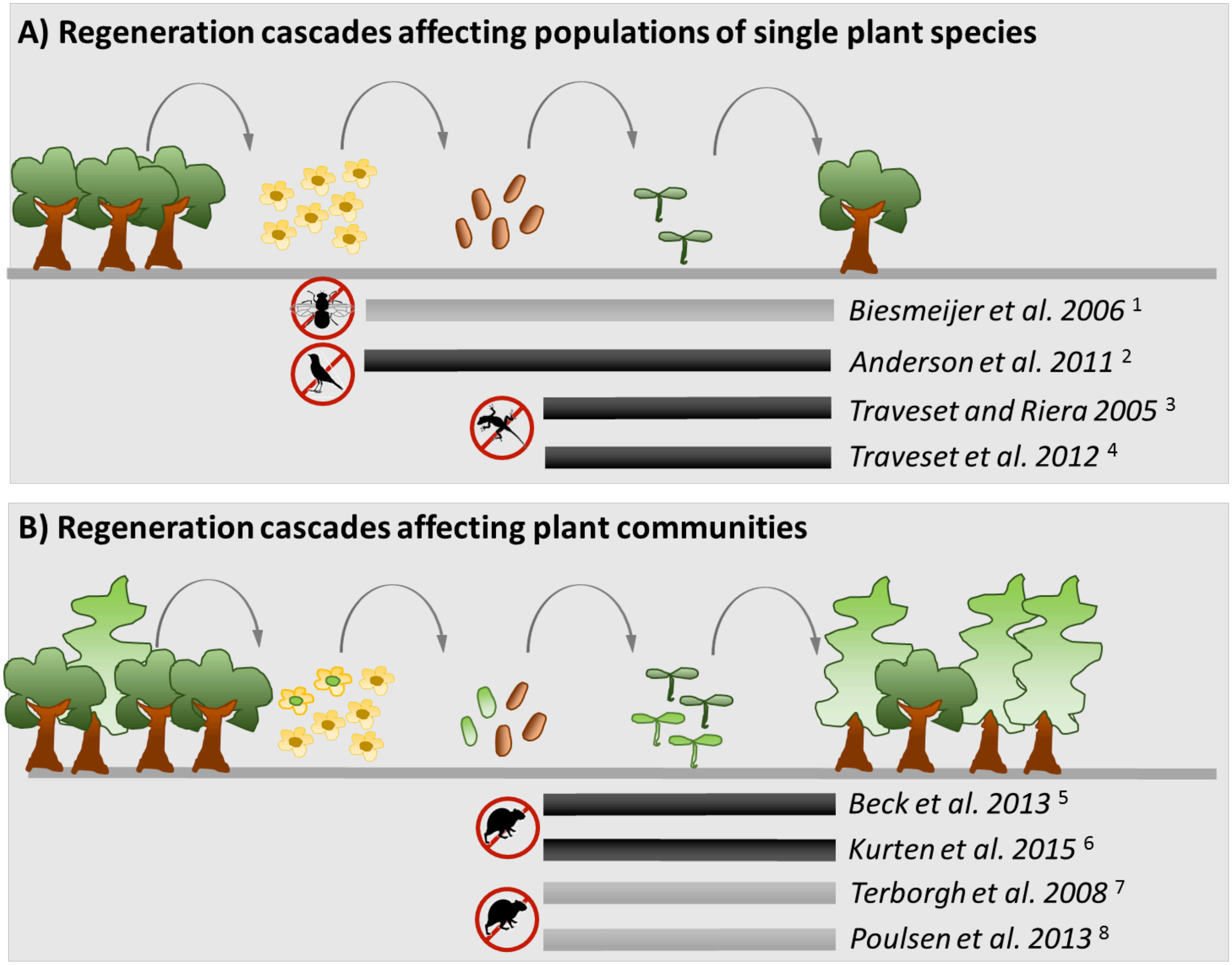

Supplementary Figure 1. Evidence of long-term cascading effects from disruptions of pollinators and seed dispersers throughout the plant regeneration cycle. (A) Negative responses of populations of single plant species and (B) plant community shifts. Black bars denote experimental exclusion and local extinction events, gray bars denote studies on regional defaunation. The length of each bar indicates the number of steps in the plant regeneration cycle covered by each study.

Supplementary Methods 1

Publication bias is a frequent phenomenon in meta-analysis, addressing that significant results are more likely to be published than non-significant results [9]. We tested for publication bias using a rank correlation test for plot asymmetry [10] with the meta package [11]. We did not find evidence for publication bias (z = -1.35, p = 0.73; Supplementary Figure 2).

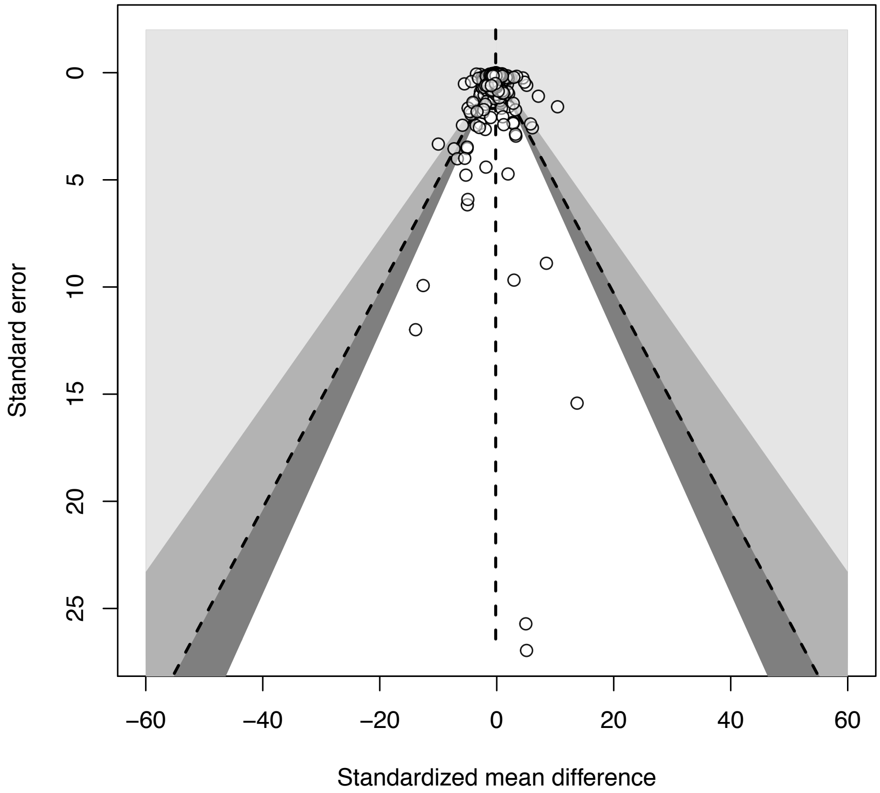

Supplementary Figure 2. Funnel-plot visualizing the absence of potential publication bias of the studies included in the meta-analysis. Points represent all 408 comparisons of plant regeneration processes between natural and human disturbed forest.

Supplementary Methods 2

We added a spatial autocovariate as fixed effect to the two models to control for spatial autocorrelation [12,13]. After including this covariate, we calculated Moran's I values from the residuals of all models based on a matrix of inverse distance weights (compiled by the geosphere package [14]) using the library ape [15]. Moran's I was not significant for any of the models.

Supplementary Methods 3

To test whether our findings are consistent with a model including a phylogenetic correlation matrix, we tested the same model compiled with the metafor package [16] with the MCMCglmm package [17], which allows testing for phylogenetic autocorrelation. We calculated a phylogenetic tree based on plant taxonomy (Supplementary Table 1) using the ape package [15] and set all branches to the length of one. We included the phylogenetic autocorrelation matrix using the "ginverse" argument. We also included the study and the species as random effects. As fixed effects, we included regeneration process, seed size, absolute latitude and the spatial autocovariate. The estimated effect sizes were consistent with the estimated effect sizes calculated by the metafor package (Supplementary Table 2).

Supplementary Table 1. Tree species included in the meta-analysis on the effect of human forest disturbance on plant regeneration processes. Taxonomic classification for all 176 plant species was obtained from The Plant List Version 1.1 (www.theplantlist.org).

Species	Family	Order	Subclass	Class
Abies guatemalensis	Pinaceae	Pinales		Coniferopsida
Abies balsamea	Pinaceae	Pinales		Coniferopsida
Acacia aroma	Leguminosae	Fabales		Magnoliopsida
Acacia atramentaria	Leguminosae	Fabales		Magnoliopsida
Acacia brachybotrya	Leguminosae	Fabales		Magnoliopsida
Acacia caven	Leguminosae	Fabales		Magnoliopsida
Acacia dealbata	Leguminosae	Fabales		Magnoliopsida
Acacia furcatispina	Leguminosae	Fabales		Magnoliopsida
Acer spicatum	Sapindaceae	Sapindales		Magnoliopsida
Aextoxicon punctatum	Aextoxicaceae	Berberidopsidales		Magnoliopsida
Alseodaphne petiolaris	Lauraceae	Laurales		Magnoliopsida
Ampelocera hottlei	Ulmaceae	Rosales		Magnoliopsida
Anacardium excelsum	Anacardiaceae	Sapindales		Magnoliopsida
Anadenanthera peregrina	Leguminosae	Fabales		Magnoliopsida
Anaxagorea dolichocarpa	Annonaceae	Magnoliales		Magnoliopsida
Antiaris toxicaria	Moraceae	Rosales		Magnoliopsida
Antrocaryon klaineanum	Anacardiaceae	Sapindales		Magnoliopsida
Apeiba glabra	Malvaceae	Malvales		Magnoliopsida
Araucaria angustifolia	Araucariaceae	Pinales		Coniferopsida
Ardisia revoluta	Primulaceae	Ericales	Asteridae	Magnoliopsida
Aristolochia chilensis	Aristolochiaceae	Piperales		Magnoliopsida
Aristotelia chilensis	Elaeocarpaceae	Oxalidales		Magnoliopsida
Aspidosperma quebracho-blanco	Apocynaceae	Gentianales		Magnoliopsida
Astrocaryum aculeatissimum	Arecaceae	Arecales		Magnoliopsida
Astrocaryum aculeatum	Arecaceae	Arecales		Magnoliopsida
Astrocaryum gratum	Arecaceae	Arecales		Magnoliopsida
Astrocaryum mexicanum	Arecaceae	Arecales		Magnoliopsida
Astrocaryum murumuru	Arecaceae	Arecales		Magnoliopsida
Astrocaryum standleyanum	Arecaceae	Arecales		Magnoliopsida
Attalea butyracea	Arecaceae	Arecales		Magnoliopsida
Attalea humilis	Arecaceae	Arecales		Magnoliopsida
Beilschmiedia assamica	Lauraceae	Laurales		Magnoliopsida
Betula papyrifera	Betulaceae	Fagales		Magnoliopsida
Billia rosea	Sapindaceae	Sapindales		Magnoliopsida
Bocageopsis multiflora	Annonaceae	Magnoliales		Magnoliopsida
Bridelia micrantha	Phyllanthaceae	Malpighiales		Magnoliopsida
Brosimum alicastrum	Moraceae	Rosales		Magnoliopsida
Buchenavia grandis	Combretaceae	Myrtales		Magnoliopsida
Bursera simaruba	Burseraceae	Sapindales		Magnoliopsida
Byrsonima sericea	Malpighiaceae	Malpighiales		Magnoliopsida
Cabralea canjerana	Meliaceae	Sapindales		Magnoliopsida
Caesalpinia gilliesii	Leguminosae	Fabales		Magnoliopsida

Caesalpinia paraguariensis	Leguminosae	Fabales		Magnoliopsida
Calophyllum brasiliense	Clusiaceae	Malpighiales		Magnoliopsida
Canarium strictum	Burseraceae	Sapindales		Magnoliopsida
Carapa guianensis	Meliaceae	Sapindales		Magnoliopsida
Carapa nicaraguensis	Meliaceae	Sapindales		Magnoliopsida
Carapa procera	Meliaceae	Sapindales		Magnoliopsida
Carapa surinamensis	Meliaceae	Sapindales		Magnoliopsida
Cariniana micrantha	Lecythidaceae	Ericales	Asteridae	Magnoliopsida
Carpinus caroliniana	Betulaceae	Fagales		Magnoliopsida
Caryocar villosum	Caryocaraceae	Malpighiales		Magnoliopsida
Ceiba aesculifolia	Malvaceae	Malvales		Magnoliopsida
Celtis africana	Cannabaceae	Rosales		Magnoliopsida
Celtis zenkeri	Cannabaceae	Rosales		Magnoliopsida
Cercidium praecox subsp. glaucum	Leguminosae	Fabales		Magnoliopsida
Cestrum parqui	Solanaceae	Solanales	Lamiidae	Magnoliopsida
Chamaedorea allenii	Arecaceae	Arecales		Magnoliopsida
Chisocheton cumingianus subsp. balansae	Meliaceae	Sapindales		Magnoliopsida
Chlorocardium rodiei	Lauraceae	Laurales		Magnoliopsida
Choerospondias axillaris	Anacardiaceae	Sapindales		Magnoliopsida
Chrysophyllum albidum	Sapotaceae	Ericales	Asteridae	Magnoliopsida
Cinnamomum triplinerve	Lauraceae	Laurales		Magnoliopsida
Clusia lechleri	Clusiaceae	Malpighiales		Magnoliopsida
Clusia sp.	Clusiaceae	Malpighiales		Magnoliopsida
Clusia sphaerocarpa	Clusiaceae	Malpighiales		Magnoliopsida
Clusia trochiformis	Clusiaceae	Malpighiales		Magnoliopsida
Cochlospermum orinocense	Bixaceae	Malvales		Magnoliopsida
Cordia alliodora	Boraginaceae	Boraginales	Lamiidae	Magnoliopsida
Cordia americana	Boraginaceae	Boraginales	Lamiidae	Magnoliopsida
Cordia bicolor	Boraginaceae	Boraginales	Lamiidae	Magnoliopsida
Cordia millenii	Boraginaceae	Boraginales	Lamiidae	Magnoliopsida
Corylus heterophylla	Betulaceae	Fagales		Magnoliopsida
Corylus mandshurica	Betulaceae	Fagales		Magnoliopsida
Cryptocarya alba	Lauraceae	Laurales		Magnoliopsida
Dendropanax arboreus	Araliaceae	Apiales	Asteridae	Magnoliopsida
Dialium guianense	Leguminosae	Fabales		Magnoliopsida
Dicorynia guianensis	Leguminosae	Fabales		Magnoliopsida
Dillenia suffruticosa	Dilleniaceae	Dilleniales		Magnoliopsida
Dinizia excelsa	Leguminosae	Fabales		Magnoliopsida
Diospyros abyssinica	Ebenaceae	Ericales	Asteridae	Magnoliopsida
Dipteryx oleifera	Leguminosae	Fabales		Magnoliopsida
Dodonaea viscosa	Sapindaceae	Sapindales		Magnoliopsida
Duckeodendron cestroides	Solanaceae	Solanales	Lamiidae	Magnoliopsida
Dysoxylum gotadhora	Meliaceae	Sapindales		Magnoliopsida
Dysoxylum malabaricum	Meliaceae	Sapindales		Magnoliopsida
Dysoxylum sp.	Meliaceae	Sapindales		Magnoliopsida
Elaeocarpus serratus	Elaeocarpaceae	Oxalidales		Magnoliopsida
Embothrium coccineum	Proteaceae	Proteales		Magnoliopsida
Eperua falcata	Leguminosae	Fabales		Magnoliopsida

Eremophila glabra	Scrophulariaceae	Lamiales	Lamiidae	Magnoliopsida
Erythroxylum pelleterianum	Erythroxylaceae	Malpighiales		Magnoliopsida
Euterpe edulis	Arecaceae	Arecales		Magnoliopsida
Euterpe precatoria	Arecaceae	Arecales		Magnoliopsida
Fagus grandifolia subsp. Mexicana	Fagaceae	Fagales		Magnoliopsida
Fagus sylvatica	Fagaceae	Fagales		Magnoliopsida
Ficus americana	Moraceae	Rosales		Magnoliopsida
Ficus aurea	Moraceae	Rosales		Magnoliopsida
Ficus lutea	Moraceae	Rosales		Magnoliopsida
Ficus racemosa	Moraceae	Rosales		Magnoliopsida
Ficus thonningii	Moraceae	Rosales		Magnoliopsida
Goupia glabra	Goupiaceae	Malpighiales		Magnoliopsida
Gustavia superba	Lecythidaceae	Ericales	Asteridae	Magnoliopsida
Harungana madagascariensis	Hypericaceae	Malpighiales		Magnoliopsida
Hedyosmum angustifolium	Chloranthaceae	Chloranthales		Magnoliopsida
Hedyosmum racemosum	Chloranthaceae	Chloranthales		Magnoliopsida
Heliconia acuminata	Heliconiaceae	Zingiberales		Magnoliopsida
Heliconia tortuosa	Heliconiaceae	Zingiberales		Magnoliopsida
Hieronyma oblonga	Phyllanthaceae	Malpighiales		Magnoliopsida
Hymenaea oblongifolia	Leguminosae	Fabales		Magnoliopsida
Jacaranda copaia	Bignoniaceae	Lamiales	Lamiidae	Magnoliopsida
Juglans mandshurica	Juglandaceae	Fagales		Magnoliopsida
Juniperus thurifera	Cupressaceae	Pinales		Coniferopsida
Lapageria rosea	Philesiaceae	Liliales		Magnoliopsida
Lecythis ampla	Lecythidaceae	Ericales	Asteridae	Magnoliopsida
Leptonychia usambarensis	Malvaceae	Malvales		Magnoliopsida
Manilkara bidentata	Sapotaceae	Ericales	Asteridae	Magnoliopsida
Manilkara zapota	Sapotaceae	Ericales	Asteridae	Magnoliopsida
Miconia albicans	Melastomataceae	Myrtales		Magnoliopsida
Miconia boliviensis	Melastomataceae	Myrtales		Magnoliopsida
Minquartia guianensis	Olacaceae	Santalales		Magnoliopsida
Myrcia paivae	Myrtaceae	Myrtales		Magnoliopsida
Myrsine coriacea	Primulaceae	Ericales	Asteridae	Magnoliopsida
Myrsine juergensenii	Primulaceae	Ericales	Asteridae	Magnoliopsida
Myrtus communis	Myrtaceae	Myrtales		Magnoliopsida
Nectandra ambigens	Lauraceae	Laurales		Magnoliopsida
Nothofagus glauca	Nothofagaceae	Fagales		Magnoliopsida
Nothofagus obliqua	Nothofagaceae	Fagales		Magnoliopsida
Ochroma pyramidale	Malvaceae	Malvales		Magnoliopsida
Ocotea floribunda	Lauraceae	Laurales		Magnoliopsida
Ocotea valeriana	Lauraceae	Laurales		Magnoliopsida
Ocotea whitei	Lauraceae	Laurales		Magnoliopsida
Oenocarpus bacaba	Arecaceae	Arecales		Magnoliopsida
Oenocarpus mapora	Arecaceae	Arecales		Magnoliopsida
Opuntia quimilo	Cactaceae	Caryophyllales		Magnoliopsida
Otoba novogranatensis	Myristicaceae	Magnoliales		Magnoliopsida
Palicourea buchtienii	Rubiaceae	Gentianales	Lamiidae	Magnoliopsida
Panopsis costaricensis	Proteaceae	Proteales		Magnoliopsida
Parashorea malaanonan	Dipterocarpaceae	Malvales		Magnoliopsida
Parkia multijuga	Leguminosae	Fabales		Magnoliopsida

Parkia pendula	Leguminosae	Fabales		Magnoliopsida
Paypayrola blanchetiana	Violaceae	Malpighiales		Magnoliopsida
Pentacme siamensis	Dipterocarpaceae	Malvales		Magnoliopsida
Phoebe sp.	Lauraceae	Laurales		Magnoliopsida
Picea glauca	Pinaceae	Pinales		Coniferopsida
Pinus ayacahuite	Pinaceae	Pinales		Coniferopsida
Pinus koraiensis	Pinaceae	Pinales		Coniferopsida
Pinus pseudostrobus	Pinaceae	Pinales		Coniferopsida
Pinus pseudostrobus var. apulcensis	Pinaceae	Pinales		Coniferopsida
Pinus sylvestris	Pinaceae	Pinales		Coniferopsida
Polyalthia simiarum	Annonaceae	Magnoliales		Magnoliopsida
Polyscias fulva	Araliaceae	Apiales	Asteridae	Magnoliopsida
Populus tremuloides	Salicaceae	Malpighiales		Magnoliopsida
Poulsenia armata	Moraceae	Rosales		Magnoliopsida
Pradosia cochlearia	Sapotaceae	Ericales	Asteridae	Magnoliopsida
Prosopis kuntzei	Leguminosae	Fabales		Magnoliopsida
Prosopis nigra	Leguminosae	Fabales		Magnoliopsida
Prunus africana	Rosaceae	Rosales		Magnoliopsida
Prunus virginiana	Rosaceae	Rosales		Magnoliopsida
Pseudolmedia spuria	Moraceae	Rosales		Magnoliopsida
Psychotria bangii	Rubiaceae	Gentianales	Lamiidae	Magnoliopsida
Psychotria mapourioides	Rubiaceae	Gentianales	Lamiidae	Magnoliopsida
Psychotria muscosa	Rubiaceae	Gentianales	Lamiidae	Magnoliopsida
Psychotria suterella	Rubiaceae	Gentianales	Lamiidae	Magnoliopsida
Psychotria tenuinervis	Rubiaceae	Gentianales	Lamiidae	Magnoliopsida
Quararibea funebris	Malvaceae	Malvales		Magnoliopsida
Quercus ilex	Fagaceae	Fagales		Magnoliopsida
Quercus mongolica	Fagaceae	Fagales		Magnoliopsida
Quercus pyrenaica	Fagaceae	Fagales		Magnoliopsida
Rhamnus ludovici-salvatoris	Rhamnaceae	Rosales		Magnoliopsida
Rhus virens	Anacardiaceae	Sapindales		Magnoliopsida
Ricinodendron heudelotii	Euphorbiaceae	Malpighiales		Magnoliopsida
Schinopsis balansae	Anacardiaceae	Sapindales		Magnoliopsida
Schinopsis lorentzii	Anacardiaceae	Sapindales		Magnoliopsida
Senna aphylla	Leguminosae	Fabales		Magnoliopsida
Senna artemisioides	Leguminosae	Fabales		Magnoliopsida
Senna multijuga	Leguminosae	Fabales		Magnoliopsida
Shorea laxa	Dipterocarpaceae	Malvales		Magnoliopsida
Sideroxylon portoricense	Sapotaceae	Ericales	Asteridae	Magnoliopsida
Simarouba amara	Simaroubaceae	Sapindales		Magnoliopsida
Sorbus aucuparia	Rosaceae	Rosales		Magnoliopsida
Sorbus torminalis	Rosaceae	Rosales		Magnoliopsida
Spondias mombin	Anacardiaceae	Sapindales		Magnoliopsida
Stenocereus quevedonis	Cactaceae	Caryophyllales		Magnoliopsida
Strychnos madagascariensis	Loganiaceae	Gentianales	Lamiidae	Magnoliopsida
Swietenia macrophylla	Meliaceae	Sapindales		Magnoliopsida
Syagrus romanzoffiana	Arecaceae	Arecales		Magnoliopsida
Symplocos coccinea	Symplocaceae	Ericales	Asteridae	Magnoliopsida
Tapirira mexicana	Anacardiaceae	Sapindales		Magnoliopsida
Ternstroemia lineata	Pentaphylacaceae	Ericales	Asteridae	Magnoliopsida

Tetragastris altissima	Burseraceae	Sapindales	Magnoliopsida
Trilepisium madagascariense	Moraceae	Rosales	Magnoliopsida
Triplaris weigeltiana	Polygonaceae	Caryophyllales	Magnoliopsida
Virola flexuosa	Myristicaceae	Magnoliales	Magnoliopsida
Virola koschnyi	Myristicaceae	Magnoliales	Magnoliopsida
Virola kwatae	Myristicaceae	Magnoliales	Magnoliopsida
Virola michelii	Myristicaceae	Magnoliales	Magnoliopsida
Virola sp.	Myristicaceae	Magnoliales	Magnoliopsida
Welfia regia	Arecaceae	Arecales	Magnoliopsida
Xymalos monospora	Monimiaceae	Laurales	Magnoliopsida
Ziziphus lotus	Rhamnaceae	Rosales	Magnoliopsida
Ziziphus mistol	Rhamnaceae	Rosales	Magnoliopsida

Supplementary Table 2. Estimated effect sizes and 95 % confidence interval (CI) based on a meta-analytic model using the MCMCglmm library [17]. We added the study and species as random effects, and a phylogenetic correlation matrix based on a taxonomic phylogenetic tree, to the model.

	**Hedge's *d***	**lower 95% CI**	**upper 95% CI**
Pollination	-0.74	-1.28	-0.27
Dispersal	-0.44	-0.80	-0.08
Predation	0.10	-0.31	0.53
Recruitment	-0.26	-0.65	0.12
Herbivory	0.20	-0.35	0.73
Seed size	-0.28	-0.65	0.05
Absolute latitude	0.01	0.07	0.11
Longitude	-0.00	-0.00	0.00
Spatial autocovariate	0.09	0.07	0.11

Supplementary References

1. Biesmeijer, J. C. *et al.* Parallel declines in pollinators and insect-pollinated plants in Britain and the Netherlands. *Science* **313,** 351–354 (2006).
2. Anderson, S. H., Kelly, D., Ladley, J. J., Molloy, S. & Terry, J. Cascading effects of bird functional extinction reduce pollination and plant density. *Science* **331,** 1068–1071 (2011).
3. Traveset, A. & Riera, N. Disruption of a plant-lizard seed dispersal system and its ecological effects on a threatened endemic plant in the Balearic Islands. *Conserv. Biol.* **19,** 421–431 (2005).
4. Traveset, A., Gonzalez-Varo, J. P. & Valido, A. Long-term demographic consequences of a seed dispersal disruption. *Proc. R. Soc. B Biol. Sci.* **279,** 3298–3303 (2012).
5. Beck, H., Snodgrass, J. W. & Thebpanya, P. Long-term exclosure of large terrestrial vertebrates: Implications of defaunation for seedling demographics in the Amazon rainforest. *Biol. Conserv.* **163,** 115–121 (2013).
6. Kurten, E. L., Wright, S. J. & Carson, W. P. Hunting alters seedling functional trait composition in a Neotropical forest. *Ecology* **96,** 1923–1932 (2015).
7. Terborgh, J. *et al.* Tree recruitment in an empty forest. *Ecology* **89,** 1757–68 (2008).
8. Poulsen, J. R., Clark, C. J. & Palmer, T. M. Ecological erosion of an Afrotropical forest and potential consequences for tree recruitment and forest biomass. *Biol. Conserv.* **163,** 122–130 (2013).
9. Koricheva, J. & Gurevitch, J. Uses and misuses of meta-analysis in plant ecology. *J. Ecol.* **102,** 828–844 (2014).
10. Begg, C. B. & Mazumdar, M. Operating characteristics of a rank correlation test for publication bias. *Biometrics* **50,** 1088–1101 (1994).
11. Schwarzer, G. meta: General Package for Meta-Analysis. R package version 4.2-0. http://CRAN.R–project.org/package=meta (2015).
12. F. Dormann, C. *et al.* Methods to account for spatial autocorrelation in the analysis of species distributional data: a review. *Ecography* **30,** 609–628 (2007).

13. Augustin, N. H., Mugglestone, M. a. & Buckland, S. T. An autologistic model for the spatial distribution of wildlife. *J. Appl. Ecol.* **33,** 339–347 (1996).

14. Hijmans, R. J. Introduction to the 'geosphere' package (Version 1.3-13). http://cran.r–project.org/web/packages/geosphere/v (2015).

15. Paradis, E., Claude, J. & Strimmer, K. APE: analyses of phylogenetics and evolution in R language. *Bioinformatics* **20,** 289–290 (2004).

16. Viechtbauer, W. Conducting meta-analysis in R with the metafor package. *J. Stat. Softw.* **36,** 1–48 (2010).

17. Hadfield, J. D. MCMC Methods for Multi-R; MCMCglmm R package. *J. Stat. Softw.* **33,** 1–22 (2010).

Supplementary Dataset - Data and list of studies included in the meta-analysis **“**Pollination and seed dispersal are the most threatened processes of plant regeneration”

Effect sizes and control variables used in the meta-analysis on the effect of human forest disturbance on plant regeneration processes. Predicted seed size for each plant species was obtained by linear regression using the species for which we had information for both seed size and seed mass. If no source publication or database for measures of seed size / seed mass is given, data were obtained from the original publication, * = seed size has been predicted based on seed mass, + = both measures of seed size and seed mass were available. Longitude and latitude are given in decimal degrees. Full references of original publications and sources of seed size / seed mass source are listed below.

**Original publication**	**Species**	**Process**	**Effect size Hedge's d**	**Sampling variance**	**Predicted seed size (cm)**		**Source publication / database for seed size /seed mass**	**Lati-tude**	**Longi-tude**
Aguilar and Galetto 2004	*Cestrum parqui*	pollination	-6.74	4.02	0.26	*	Funes et al. 2009	-31.26	-64.28
Aguirre and Dirzo 2008	*Astrocaryum mexicanum*	pollination	1.18	1.05	2.43	*	TRY	19.54	-96.93
Aizen and Feinsinger 1994	*Acacia aroma*	pollination	-0.72	1.44	0.6			-26.83	-65.33
Aizen and Feinsinger 1994	*Acacia atramentaria*	pollination	0.61	1.38	0.69	*	Funes et al. 2009	-26.83	-65.33
Aizen and Feinsinger 1994	*Acacia furcatispina*	pollination	0.56	1.36	0.65	*	Funes et al. 2009	-26.83	-65.33
Aizen and Feinsinger 1994	*Caesalpinia gilliesii*	pollination	-0.41	1.31	1		Encyclopedia of Life	-26.83	-65.33
Aizen and Feinsinger 1994	*Cercidium praecox subsp. glaucum*	pollination	-0.82	1.35	0.48	*	TRY	-26.83	-65.33
Aizen and Feinsinger 1994	*Opuntia quimilo*	pollination	-1.96	2.65	0.45		Reyes-Agüero et al. 2006	-26.83	-65.33
Aizen and Feinsinger 1994	*Prosopis nigra*	pollination	-0.97	1.4	0.75	*	TRY	-26.83	-65.33
Aizen and Feinsinger 1994	*Senna aphylla*	pollination	-1.22	1.79	0.32	*	Funes et al. 2009	-26.83	-65.33
Albrecht et al. 2012	*Sorbus aucuparia*	dispersal	-0.16	0.33	0.22	*	TRY	50.8	8.73
Albrecht et al. 2012	*Sorbus aucuparia*	dispersal	-2.34	0.66	0.22	*	TRY	50.8	8.73
Aliaga-Rossel and Fragoso 2014	*Astrocaryum gratum*	predation	-5.05	3.47	10.00		Aliaga-Rossel 2011	-13.33	-68.17
Alvarez-Aquino et al. 2004	*Carpinus caroliniana*	recruitment	-1.94	1.48	0.48	*	TRY	19.34	-96.99
Alvarez-Aquino et al. 2004	*Fagus grandifolia subsp. Mexicana*	recruitment	-2.97	2.56	1.01	*	Greene & Johnson 1994	19.34	-96.99

Alvarez-Aquino et al. 2004	*Symplocos coccinea*	recruitment	-0.96	0.86	1.36	*	Saldaña-Acosta et al. 2008	19.34	-96.99
Andreazzi et al. 2012	*Attalea humilis*	dispersal	-1.73	0.34	4.42			-23.15	-45.03
Andreazzi et al. 2012	*Attalea humilis*	pollination	1.37	0.3	4.42			-23.15	-45.03
Andreazzi et al. 2012	*Attalea humilis*	predation	-1.09	0.28	4.42			-23.15	-45.03
Andreazzi et al. 2012	*Attalea humilis*	predation	0.07	0.23	4.42			-23.15	-45.03
Andreazzi et al. 2012	*Attalea humilis*	predation	-1.37	0.3	4.42			-23.15	-45.03
Anzures-Dadda et al. 2011	*Brosimum alicastrum*	recruitment	0.56	1.31	1.7	*	TRY	17.78	-93.22
Anzures-Dadda et al. 2011	*Cordia alliodora*	recruitment	0.54	1.29	0.42	*	TRY	17.78	-93.22
Anzures-Dadda et al. 2011	*Dialium guianense*	recruitment	-1.85	4.41	1.01	*	TRY	17.78	-93.22
Anzures-Dadda et al. 2011	*Manilkara zapota*	recruitment	-0.46	1.21	1.99	+	TRY, OFarrill et al. 2011	17.78	-93.22
Anzures-Dadda et al. 2011	*Nectandra ambigens*	recruitment	0.14	1.02	1.96	*	TRY	17.78	-93.22
Anzures-Dadda et al. 2011	*Quararibea funebris*	recruitment	-0.14	1.02	1.57	*	TRY	17.78	-93.22
Anzures-Dadda et al. 2011	*Spondias mombin*	recruitment	0.81	1.66	2.21	*	TRY	17.78	-93.22
Asbjornsen et al. 2004	*Dodonaea viscosa*	recruitment	-0.23	0.68	0.31	+	Burrows et al. 1995	17	-96
Asbjornsen et al. 2004	*Pinus pseudostrobus var. apulcensis*	recruitment	0.15	0.67	0.47	*	TRY	17	-96
Asbjornsen et al. 2004	*Rhus virens*	recruitment	-0.01	0.67	0.49	*	TRY	17	-96
Ashworth et al. 2015	*Acacia caven*	recruitment	-2.22	1.72	0.76	*	TRY	-29.75	-64.00
Ashworth and Marti 2011	*Acacia caven*	pollination	0.08	0.67	0.72	*	TRY	-29.75	-64
Athayde and Morellato 2014	*Anadenanthera peregrina*	pollination	-3.48	0.06	1		Encyclopedia of Life	-22.18	-47.87
Athayde and Morellato 2014	*Anadenanthera peregrina*	recruitment	-0.4	0.1	1		Encyclopedia of Life	-22.18	-47.87
Avalos et al. 2013	*Euterpe precatoria*	recruitment	-0.6	1.36	1.1		Homeier et al. 2002	10.32	-83.87
Avalos et al. 2013	*Euterpe precatoria*	recruitment	-0.05	1	1.1		Homeier et al. 2002	10.32	-83.87
Babweteera & Brown 2009	*Celtis zenkeri*	dispersal	-1.17	0.96	1			0.92	31.69
Babweteera & Brown 2009	*Chrysophyllum albidum*	dispersal	-1.42	1.1	2.4	+	Oyelade et al. 2005	0.92	31.69
Babweteera & Brown 2009	*Cordia millenii*	dispersal	-2.07	1.58	4			0.92	31.69
Babweteera & Brown 2009	*Ricinodendron heudelotii*	dispersal	-0.81	0.81	1.88	*	TRY	0.92	31.69
Babweteera 2009	*Cordia millenii*	dispersal	-0.7	0.77	4			0.92	31.69
Bagchi et al. 2011	*Parashorea malaanonan*	predation	0	0.2	1.98	*	TRY	4.97	118.8
Barbeta et al. 2011	*Fagus sylvatica*	recruitment	-3.12	0.26	0.9	*	TRY	41.79	2.41
Barrera Zambrano et al. 2008	*Apeiba glabra*	dispersal	-1.79	0.53	0.25			-3.41	-70.16

Barrera Zambrano et al. 2008	*Apeiba glabra*	dispersal	-0.29	0.34	0.25			-3.41	-70.16
Barrera Zambrano et al. 2008	*Hymenaea oblongifolia*	dispersal	0.79	0.37	1.95			-3.41	-70.16
Barrera Zambrano et al. 2008	*Hymenaea oblongifolia*	dispersal	-0.14	0.02	1.95			-3.41	-70.16
Beckman and Muller-Landau 2007	*Cordia bicolor*	dispersal	-0.61	0.36	0.77	*		9.12	-79.77
Beckman and Muller-Landau 2007	*Cordia bicolor*	predation	1.28	0.43	0.77	*		9.12	-79.77
Beckman and Muller-Landau 2007	*Oenocarpus mapora*	dispersal	-3.92	1.42	1.93	*		9.12	-79.77
Beckman and Muller-Landau 2007	*Oenocarpus mapora*	predation	-4.59	1.82	1.93	*		9.12	-79.77
Boissier et al. 2014	*Virola michelii*	dispersal	-1.81	0.17	2.00		Forget and Cuijpers 2008	4.08	-52.67
Boissier et al. 2014	*Virola sp.*	dispersal	-1.16	0.12	2.80			4.08	-52.67
Bose et al. 2014	*Populus tremuloides*	recruitment	3.27	2.96	0.10	+		49.43	-79.31
Bose et al. 2014	*Betula papyrifera*	recruitment	3.20	2.87	0.45	+	Encyclopedia of Life	49.43	-79.31
Bose et al. 2014	*Abies balsamea*	recruitment	-0.36	0.69	1.20	+	Encyclopedia of Life, St-Denis et al. 2013	49.43	-79.31
Bose et al. 2014	*Acer spicatum*	recruitment	-4.95	5.92	2.50		Encyclopedia of Life	49.43	-79.31
Braun and Gottsberger 2012	*Anaxagorea dolichocarpa*	pollination	-0.09	0.67	1.6		Encyclopedia of Life	-7.79	-34.99
Braun and Gottsberger 2012	*Anaxagorea dolichocarpa*	pollination	-0.24	0.68	1.6		Encyclopedia of Life	-7.79	-34.99
Braun et al. 2012	*Paypayrola blanchetiana*	pollination	-2.36	1.86	0.9		Encyclopedia of Life	-7.76	-34.99
Broadhust and Young 2006	*Acacia dealbata*	pollination	-0.5	0.12	0.36	*	TRY	-35.48	149.01
Brodie et al. 2009	*Choerospondias axillaris*	dispersal	-3.29	1.76	2.03	*	TRY	17.12	100.1
Brum et al. 2008	*Oenocarpus bacaba*	recruitment	-2.35	0.37	2.5			-2.5	-60
Brum et al. 2008	*Oenocarpus bacaba*	recruitment	0.82	0.21	2.5			-2.5	-60
Bruna 2002	*Heliconia acuminata*	recruitment	-3.53	2.47	0.7			-2.5	-60
Bruna 2002	*Heliconia acuminata*	recruitment	-5.27	4.78	0.7			-2.5	-60
Camison et al. 2015	*Quercus pyrenaica*	recruitment	1.02	0.97	3.10	+	Encyclopedia of Life, Quero et al. 2007	40.56	-5.95
Campana Camargo et al. 2002	*Buchenavia grandis*	recruitment	1.84	0.89	2.19	*	TRY	-2.55	-60

Campana Camargo et al. 2002	*Buchenavia grandis*	recruitment	0.26	0.51	2.19	*	TRY	-2.55	-60
Campana Camargo et al. 2002	*Cariniana micrantha*	recruitment	-1.69	0.83	0.89	*		-2.55	-60
Campana Camargo et al. 2002	*Cariniana micrantha*	recruitment	-0.77	0.57	0.89	*		-2.55	-60
Campana Camargo et al. 2002	*Caryocar villosum*	recruitment	2.82	1.43	2.96	*		-2.55	-60
Campana Camargo et al. 2002	*Caryocar villosum*	recruitment	8.49	8.89	2.96	*		-2.55	-60
Campana Camargo et al. 2002	*Cochlospermum orinocense*	recruitment	0.48	0.53	0.5	*		-2.55	-60
Campana Camargo et al. 2002	*Cochlospermum orinocense*	recruitment	-0.46	0.52	0.5	*		-2.55	-60
Campana Camargo et al. 2002	*Dinizia excelsa*	recruitment	-0.86	0.59	0.94	*		-2.55	-60
Campana Camargo et al. 2002	*Jacaranda copaia*	recruitment	0.59	0.54	0.29	*	TRY	-2.55	-60
Campana Camargo et al. 2002	*Ochroma pyramidale*	recruitment	0.19	0.5	0.35	*	TRY	-2.55	-60
Campana Camargo et al. 2002	*Parkia multijuga*	recruitment	-2.15	1.04	3.14	*		-2.55	-60
Campana Camargo et al. 2002	*Parkia multijuga*	recruitment	-5.48	4	3.14	*		-2.55	-60
Campana Camargo et al. 2002	*Parkia pendula*	recruitment	-1.24	0.68	0.69	*	TRY	-2.55	-60
Campana Camargo et al. 2002	*Simarouba amara*	recruitment	0.36	0.52	0.85	*	TRY	-2.55	-60
Campana Camargo et al. 2002	*Triplaris weigeltiana*	recruitment	0.76	0.57	0.75	*		-2.55	-60
Campana Camargo et al.	*Triplaris weigeltiana*	recruitment	-0.39	0.52	0.75	*		-2.55	-60

2002									
Chacoff et al. 2004	*Acacia aroma*	pollination	0.23	0.51	0.6		Markl et al. 2012	-26.19	-62.1
Chapman et al. 2003	*Diospyros abyssinica*	predation	-1.08	0.09	0.75		Markl et al. 2012	0.45	30.43
Chinchilla 2009	*Billia rosea*	predation	-1.45	0.46	6			10.3	-84.8
Chinchilla 2009	*Ocotea valeriana*	predation	-2.18	0.62	6.5			10.3	-84.8
Chinchilla 2009	*Ocotea whitei*	predation	-1.84	0.54	4	+	TRY	10.3	-84.8
Chinchilla 2009	*Panopsis costaricensis*	predation	-2.46	0.7	5			10.3	-84.8
Christianini and Oliviera 2013	*Erythroxylum pelleterianum*	predation	0.56	0.24	0.55	*	Christianini et al 2007	-22.2	-47.85
Christianini and Oliviera 2013	*Erythroxylum pelleterianum*	recruitment	-0.14	0.23	0.55	*	Christianini et al 2007	-22.2	-47.85
Cordeiro and Howe 2003	*Leptonychia usambarensis*	dispersal	-0.98	0.18	0.14	+	TRY	-4	38
Cordeiro and Howe 2003	*Leptonychia usambarensis*	dispersal	-0.91	0.18	0.14	+	TRY	-4	38
Cordeiro and Howe 2003	*Leptonychia usambarensis*	dispersal	-0.61	0.17	0.14	+	TRY	-4	38
Cordeiro et al. 2009	*Leptonychia usambarensis*	predation	-0.53	0.53	0.14	+	TRY	-4	38
Cordeiro et al. 2009	*Leptonychia usambarensis*	recruitment	0.28	0.51	0.14	+	TRY	-4	38
Cramer et al. 2007a	*Bocageopsis multiflora*	dispersal	0.38	0.52	0.5	+	TRY, Markl et al. 2012	-2.5	-60
Cramer et al. 2007a	*Bocageopsis multiflora*	dispersal	-0.24	0.51	0.5	+	TRY, Markl et al. 2013	-2.5	-60
Cramer et al. 2007a	*Duckeodendron cestroides*	dispersal	-2.79	0.9	5.9			-2.5	-60
Cramer et al. 2007a	*Duckeodendron cestroides*	dispersal	-1.98	0.64	5.9			-2.5	-60
Cramer et al. 2007a	*Duckeodendron cestroides*	recruitment	0.09	0.37	5.9			-2.5	-60
Cramer et al. 2007a	*Duckeodendron cestroides*	recruitment	-1.28	0.48	5.9			-2.5	-60
Cramer et al. 2007b	*Duckeodendron cestroides*	dispersal	-0.42	0.38	5.9			-2.5	-60
Cramer et al. 2007b	*Duckeodendron cestroides*	dispersal	-0.66	0.4	5.9			-2.5	-60
Cunningham 2000	*Acacia brachybotrya*	pollination	-0.77	0.57	0.45	*	TRY	-33.51	146.17
Cunningham 2000	*Eremophila glabra*	pollination	-1.54	0.77	0.7	*	TRY	-33.52	146.18
Cunningham 2000	*Senna artemisioides*	pollination	0.79	0.57	0.4	*	TRY	-33.53	146.19
daSilva et al. 2011	*Syagrus romanzoffiana*	predation	-1.03	0.23	2.5	+	TRY	-28.2	-48.5
daSilva et al. 2011	*Syagrus romanzoffiana*	recruitment	-0.22	0.2	2.5	+	TRY	-28.2	-48.5
Dausmann et al. 2008	*Strychnos madagascariensis*	recruitment	0.66	1.43	1.9	*		-20.07	44.67
del-Val et al. 2007	*Aextoxicon punctatum*	recruitment	0	0.5	1.11	*	TRY	-30.67	-71.5

Donatti et al. 2009	*Astrocaryum aculeatissimum*	dispersal	-1.22	0.41	4.27	+	Galetti et al 2010	-24	-46.39
Donatti et al. 2009	*Astrocaryum aculeatissimum*	predation	-1.85	0.46	4.27	+	Galetti et al 2010	-24	-46.39
Donatti et al. 2009	*Astrocaryum aculeatissimum*	recruitment	-1.74	0.47	4.27	+	Galetti et al 2010	-24	-46.39
Donoso et al. 2003	*Nothofagus glauca*	predation	4.47	0.24	1.8	+	TRY	-36.58	-72.5
Donoso et al. 2003	*Nothofagus glauca*	predation	4.75	0.44	1.8	+	TRY	-36.58	-72.5
Donoso et al. 2003	*Nothofagus obliqua*	predation	3.41	0.17	0.6	+	TRY	-36.58	-72.5
Donoso et al. 2003	*Nothofagus obliqua*	predation	1.42	0.16	0.6	+	TRY	-36.58	-72.5
Dunley et al. 2009	*Byrsonima sericea*	pollination	2.82	2.37	0.5		Encyclopedia of Life	-22.94	-42.05
Dunley et al. 2009	*Byrsonima sericea*	pollination	0.51	0.72	0.5		Encyclopedia of Life	-22.94	-42.05
Fadini et al. 2009	*Euterpe edulis*	dispersal	-0.5	0.14	1.5	*	Galetti et al 2010	-23.56	-45.29
Fadini et al. 2009	*Euterpe edulis*	dispersal	0.01	0.13	1.5	*	Galetti et al 2010	-23.56	-45.29
Fadini et al. 2009	*Euterpe edulis*	predation	5.12	0.59	1.5	*	Galetti et al 2010	-23.56	-45.29
Farwig et al 2008	*Prunus africana*	predation	0.11	0.45	0.81	+	TRY	0.29	34.79
Farwig et al. 2006	*Prunus africana*	dispersal	0.77	0.16	0.81	+	TRY	0.29	34.79
Farwig et al. 2006	*Prunus africana*	dispersal	0.38	0.15	0.81	+	TRY	0.29	34.79
Figueroa-Esquivel et al. 2009	*Dendropanax arboreus*	dispersal	0.11	0.4	0.45	+	TRY, Graham et al. 2002	18.6	-95.06
Figueroa-Esquivel et al. 2009	*Dendropanax arboreus*	dispersal	-0.89	0.12	0.45	+	TRY, Graham et al. 2002	18.6	-95.06
Fleury and Galetti 2006	*Syagrus romanzoffiana*	predation	1.14	0.95	2.50	+	TRY, daSilva et al. 2011	-22.66	-48.18
Fleury and Galetti 2006	*Syagrus romanzoffiana*	predation	-0.03	0.67	2.50	+	TRY, daSilva et al. 2011	-22.66	-48.18
Fleury and Galetti 2004	*Euterpe edulis*	predation	-0.21	0.68	1.5	*	Galetti et al 2010	-22.41	-48.95
Fleury and Galetti 2004	*Syagrus romanzoffiana*	predation	1.36	1.06	2.5	+	TRY, daSilva et al. 2011	-22.41	-48.95
Forget and Cuijpers 2008	*Virola kwatae*	predation	0.61	0.54	2.8	+		5.02	-55.57
Forget and Cuijpers 2008	*Virola kwatae*	predation	0.87	0.59	2.8	+		5.02	-55.57
Forget and Jansen 2007	*Carapa surinamensis*	dispersal	-0.38	0.46	3.75			-5.35	-54.45
Forget and Jansen 2007	*Carapa surinamensis*	predation	-0.65	0.49	3.75			-5.35	-54.45
Forget and Jansen 2007	*Carapa surinamensis*	predation	-0.45	0.47	3.75			-5.35	-54.45
Forget et al. 2001	*Carapa procera*	recruitment	-0.1	0.1	4	+		5.27	-52.92
Forget et al. 2001	*Manilkara bidentata*	recruitment	-1.44	0.13	2.25			5.27	-52.92
Forget et al. 2001	*Pradosia cochlearia*	recruitment	0.04	0.1	3	+		5.27	-52.92
Franceschinelli et al. 2015	*Cabralea canjerana*	pollination	0.95	0.86	2.80	*		-22.71	-45.93
Franceschinelli et al. 2015	*Cabralea canjerana*	pollination	-0.82	0.81	2.80	*		-22.71	-45.93

Galetti et al. 2015	*Euterpe edulis*	predation	-0.04	0.58	1.54	+	Galetti et al 2010	-25.18	-47.98
Galetti at al. 2006	*Astrocaryum aculeatissimum*	dispersal	-5.02	6.17	4.27	+	Galetti et al 2010	-23.15	-45.03
Galetti at al. 2006	*Astrocaryum aculeatissimum*	predation	5.1	26.96	4.27	+	Galetti et al 2010	-23.15	-45.03
Galetti at al. 2006	*Astrocaryum aculeatissimum*	predation	-1.05	2.09	4.27	+	Galetti et al 2010	-23.15	-45.03
Galetti at al. 2006	*Astrocaryum aculeatissimum*	predation	-4.31	7.6	4.27	+	Galetti et al 2010	-23.15	-45.03
Galetti at al. 2006	*Astrocaryum aculeatissimum*	recruitment	0.5	0.72	4.27	+	Galetti et al 2010	-23.15	-45.03
Galetti at al. 2006	*Astrocaryum aculeatissimum*	recruitment	-0.51	0.72	4.27	+	Galetti et al 2010	-23.15	-45.03
Gallegos et al. 2014	*Clusia trochiformis*	predation	-0.37	0.34	0.39	+	Personal comm.	-16.4	-67.52
Gallegos et al. 2014	*Clusia trochiformis*	predation	0.54	0.35	0.39	+	Personal comm.	-16.4	-67.52
Gallegos et al. 2015	*Clusia lechleri*	recruitment	-0.04	0.2	0.41	+	Personal comm.	-16.41	-67.53
Gallegos et al. 2015	*Clusia lechleri*	recruitment	0.42	0.21	0.41	+	Personal comm.	-16.41	-67.53
Gallegos et al. 2015	*Clusia sphaerocarpa*	recruitment	0.35	0.2	0.64	+	Personal comm.	-16.41	-67.53
Gallegos et al. 2015	*Clusia sphaerocarpa*	recruitment	-0.72	0.22	0.64	+	Personal comm.	-16.41	-67.53
Gallegos et al. 2015	*Clusia sphaerocarpa*	recruitment	-0.38	0.2	0.64	+	Personal comm.	-16.41	-67.53
Gallegos et al. 2015	*Clusia trochiformis*	recruitment	0.34	0.2	0.39	+	Personal comm.	-16.41	-67.53
Gallegos et al. 2015	*Clusia trochiformis*	recruitment	0.31	0.2	0.39	+	Personal comm.	-16.41	-67.53
Gallegos et al. 2015	*Clusia trochiformis*	recruitment	0.33	0.2	0.39	+	Personal comm.	-16.41	-67.53
Gallegos Ayala 2014	*Hedyosmum angustifolium*	recruitment	-0.53	0.21	0.31	+	Personal comm.	-16.41	-67.53
Gallegos Ayala 2014	*Hedyosmum racemosum*	recruitment	0.44	0.21	0.3	+	Personal comm.	-16.41	-67.53
Gallegos Ayala 2014	*Miconia boliviensis*	recruitment	-0.12	0.2	0.09	+	Personal comm.	-16.41	-67.53
Gallegos Ayala 2014	*Myrcia paivae*	recruitment	-0.22	0.2	0.55	+	Personal comm.	-16.41	-67.53
Gallegos Ayala 2014	*Myrsine coriacea*	recruitment	0.22	0.2	0.26	+	Personal comm.	-16.41	-67.53
Gallegos Ayala 2014	*Palicourea buchtienii*	recruitment	-0.36	0.2	0.29	+	Personal comm.	-16.41	-67.53
Gallegos Ayala 2014	*Psychotria bangii*	recruitment	0.29	0.2	0.35	+	Personal comm.	-16.41	-67.53
Ghazoul and McLeish 2001	*Anacardium excelsum*	pollination	-0.72	0.49	2.24	*	TRY	10.45	-85.1
Ghazoul and McLeish 2001	*Anacardium excelsum*	pollination	-1.77	0.42	2.24	*	TRY	10.45	-85.1
Ghazoul and McLeish 2001	*Pentacme siamensis*	pollination	-1.23	0.28	2		Marod et al. 2002	15.6	99.33
Ghazoul et al. 1998	*Pentacme siamensis*	pollination	-1.19	0.27	2		Marod et al. 2002	15.6	99.33
Ghazoul et al. 1998	*Pentacme siamensis*	predation	-0.71	0.1	2		Marod et al. 2002	15.6	99.33
Ghazoul et al. 1998	*Pentacme siamensis*	predation	-0.17	0.1	2		Marod et al. 2002	15.6	99.33
Gonzalez-Di Pierro et al.	*Ampelocera hottlei*	recruitment	-0.69	0.77	1.2	+	TRY	16.1	-90.88

2011									
Gonzalez-Varo 2010	*Myrtus communis*	dispersal	-0.29	0.46	0.36	*		36.91	-6.25
Gonzalez-Varo et al. 2012	*Myrtus communis*	pollination	0.61	0.54	0.36	*		36.91	-6.25
Gonzalez-Varo et al. 2012	*Myrtus communis*	predation	-0.49	0.53	0.36	*		36.91	-6.25
Gonzalez-Varo et al. 2012	*Myrtus communis*	recruitment	-0.77	0.57	0.36	*		36.91	-6.25
Gonzalez-Varo et al. 2012	*Myrtus communis*	recruitment	-1.44	0.65	0.36	*		36.91	-6.25
Graham et al. 2002	*Bursera simaruba*	dispersal	0.37	0.22	0.75	+	TRY	19.54	-96.93
Graham et al. 2002	*Bursera simaruba*	dispersal	-0.02	0.22	0.75	+	TRY	19.54	-96.93
Graham et al. 2002	*Dendropanax arboreus*	dispersal	0.06	0.36	0.45	+	TRY	19.54	-96.93
Graham et al. 2002	*Dendropanax arboreus*	dispersal	0.72	0.39	0.45	+	TRY	19.54	-96.93
Guariguata et al. 2000	*Carapa nicaraguensis*	dispersal	-1.39	0.17	4.5			16.1	-90.88
Guariguata et al. 2000	*Lecythis ampla*	dispersal	-1.08	0.16	5.25			16.1	-90.88
Guariguata et al. 2000	*Minquartia guianensis*	dispersal	-0.05	0.13	2.2			16.1	-90.88
Guariguata et al. 2000	*Otoba novogranatensis*	dispersal	0.42	0.14	2.36			16.1	-90.88
Guariguata et al. 2000	*Virola koschnyi*	dispersal	2.24	0.23	2			16.1	-90.88
Guariguata et al. 2000	*Welfia regia*	dispersal	0.34	0.14	2.28	+	Beckmann & Muller-Landau 2007	16.1	-90.88
Guariguata et al. 2002	*Carapa guianensis*	dispersal	0	0.13	4.5	+	TRY	16.1	-90.88
Guariguata et al. 2002	*Dipteryx panamensis*	dispersal	2.43	0.25	7	+	Beckmann & Mueller-Landau 2007	16.1	-90.88
Guariguata et al. 2002	*Dipteryx panamensis*	dispersal	0.5	0.14	7	+	Beckmann & Mueller-Landau 2007	16.1	-90.88
Guariguata et al. 2002	*Dipteryx panamensis*	predation	7.12	1.1	7	+	Beckmann & Mueller-Landau 2007	16.1	-90.88
Guerrero and Bustamente 2009	*Aristolochia chilensis*	recruitment	1	0.08	0.3	*	TRY	-35.97	-72.7
Guerrero and Bustamente 2009	*Aristolochia chilensis*	recruitment	1.95	0.26	0.3	*	TRY	-35.97	-72.7
Guerrero and Bustamente 2009	*Aristolochia chilensis*	recruitment	1.62	0.23	0.3	*	TRY	-35.97	-72.7
Guerrero and Bustamente	*Cryptocarya alba*	recruitment	-0.04	0.07	1.51	*	TRY	-35.97	-72.7

2009									
Guerrero and Bustamente 2009	*Cryptocarya alba*	recruitment	-1.15	0.2	1.51	*	TRY	-35.97	-72.7
Guerrero and Bustamente 2009	*Cryptocarya alba*	recruitment	-1.65	0.23	1.51	*	TRY	-35.97	-72.7
Guerrero and Bustamente 2009	*Nothofagus glauca*	recruitment	-0.24	0.07	1.8	+	TRY, Donoso et al. 2003	-35.97	-72.7
Guerrero and Bustamente 2009	*Nothofagus glauca*	recruitment	0.39	0.17	1.8	+	TRY, Donoso et al. 2003	-35.97	-72.7
Guerrero and Bustamente 2009	*Nothofagus obliqua*	recruitment	0.94	0.08	0.6	+	TRY, Donoso et al. 2003	-35.97	-72.7
Guerrero and Bustamente 2009	*Nothofagus obliqua*	recruitment	0.35	0.17	0.6	+	TRY, Donoso et al. 2003	-35.97	-72.7
Guerrero and Bustamente 2009	*Nothofagus obliqua*	recruitment	0.69	0.18	0.6	+	TRY, Donoso et al. 2003	-35.97	-72.7
Guiterrez-Granados et al. 2011	*Swietenia macrophylla*	predation	0.41	0.7	1.32	*	TRY	19.5	-88.17
Guiterrez-Granados et al. 2011	*Swietenia macrophylla*	predation	-0.72	0.78	1.32	*	TRY	19.5	-88.17
Guiterrez-Granados et al. 2011	*Swietenia macrophylla*	predation	0.05	0.67	1.32	*	TRY	19.5	-88.17
Guiterrez-Granados et al. 2011	*Swietenia macrophylla*	recruitment	0.62	0.75	1.32	*	TRY	19.5	-88.17
Guiterrez-Granados et al. 2011	*Swietenia macrophylla*	recruitment	0.75	0.79	1.32	*	TRY	19.5	-88.17
Gutierrez-Granados and Dirzo 2010	*Manilkara zapota*	dispersal	-1.27	0.16	1.99	+	TRY, OFarrill et al. 2011	19.5	-88.17
Gutierrez-Granados and Dirzo 2010	*Manilkara zapota*	recruitment	1.81	0.15	1.99	+	TRY, OFarrill et al. 2011	19.5	-88.17
Hanson et al. 2006	*Dipteryx panamensis*	predation	-0.61	0.26	7	+	Beckmann & Muller-Landau 2007, Guariguata	10.15	-83.99

							et al. 2002		
Hanson et al. 2006	*Dipteryx panamensis*	recruitment	0.48	0.07	7	+	Beckmann & Muller-Landau 2007, Guariguata et al. 2002	10.15	-83.99
Herrerias-Diego et al. 2006	*Ceiba aesculifolia*	pollination	0.37	0.05	0.8	+	TRY, Markl et al. 2012	19.5	-105.05
Hoebee et al. 2007	*Sorbus torminalis*	pollination	-1.18	0.07	0.59	*	TRY	47.54	8.645
Holbrook and Loiselle 2009	*Virola flexuosa*	dispersal	-4.89	1.65	1.5			0.65	-76.28
Holbrook and Loiselle 2009	*Virola flexuosa*	dispersal	-0.2	0.36	1.5			0.65	-76.28
Holl and Lulow 1997	*Ardisia revoluta*	predation	0.54	0.35	0.51	+		8.95	-82.83
Holl and Lulow 1997	*Ardisia revoluta*	predation	1.83	0.54	0.51	+		8.95	-82.83
Holl and Lulow 1997	*Calophyllum brasiliense*	predation	-1.19	0.42	1.92	+		8.95	-82.83
Holl and Lulow 1997	*Chamaedorea allenii*	predation	-0.76	0.37	0.77	+		8.95	-82.83
Holl and Lulow 1997	*Chamaedorea allenii*	predation	-0.59	0.35	0.77	+		8.95	-82.83
Holl and Lulow 1997	*Cinnamomum triplinerve*	predation	1.06	0.4	1.57	+		8.95	-82.83
Holl and Lulow 1997	*Cinnamomum triplinerve*	predation	-1.36	0.44	1.57	+		8.95	-82.83
Holl and Lulow 1997	*Heliconia tortuosa*	predation	-2.49	0.71	0.93	+		8.95	-82.83
Holl and Lulow 1997	*Heliconia tortuosa*	predation	-0.44	0.34	0.93	+		8.95	-82.83
Holl and Lulow 1997	*Hieronyma oblonga*	predation	-1.25	0.43	0.59	+		8.95	-82.83
Holl and Lulow 1997	*Hieronyma oblonga*	predation	6.09	2.57	0.59	+		8.95	-82.83
Holl and Lulow 1997	*Ocotea floribunda*	predation	-0.33	0.34	1.68	+		8.95	-82.83
Holl and Lulow 1997	*Pseudolmedia spuria*	predation	0.98	0.39	1.63	+		8.95	-82.83
Holl and Lulow 1997	*Sideroxylon portoricense*	predation	-1.9	0.55	1.55	+		8.95	-82.83
Iob and Vieira 2008	*Araucaria angustifolia*	predation	-0.34	0.1	3.06	*	TRY	-29.49	-50.21
Ismail et al. 2014	*Dysoxylum malabaricum*	recruitment	-1.66	0.82	2.19	*	TRY	12.91	75.59
Ismail et al. 2014	*Dysoxylum malabaricum*	recruitment	-0.28	0.51	2.19	*	TRY	12.91	75.59
Jorge and Howe 2009	*Astrocaryum aculeatum*	dispersal	0.96	0.08	3.6	+	TRY, Markl et al. 2012	-2.44	-60.04
Jorge and Howe 2009	*Astrocaryum aculeatum*	dispersal	0.53	0.12	3.6	+	TRY, Markl et al. 2012	-2.44	-60.04
Jorge and Howe 2009	*Astrocaryum aculeatum*	predation	0.96	0.08	3.6	+	TRY, Markl et al. 2012	-2.44	-60.04
Khan et al. 2005	*Elaeocarpus serratus*	dispersal	-4.4	1.94	1.7	+		27.14	95.5
Khan et al. 2005	*Elaeocarpus serratus*	dispersal	0.18	0.4	1.7	+		27.14	95.5
Khan et al. 2005	*Elaeocarpus serratus*	pollination	-1.49	0.58	1.7	+		27.14	95.5

Khan et al. 2005	*Elaeocarpus serratus*	predation	13.74	15.42	1.7	+		27.14	95.5
Kirika et al. 2008	*Ficus thonningii*	dispersal	-0.82	0.18	0.12	+	TRY	0.25	34.86
Kirika et al. 2008	*Ficus thonningii*	dispersal	-0.51	0.17	0.12	+	TRY	0.25	34.86
Lefevre and Rodd 2009	*Psychotria mapourioides*	dispersal	-0.12	0.17	0.25			11.28	-60.62
Lefevre and Rodd 2009	*Psychotria muscosa*	dispersal	-0.41	0.17	0.3			11.28	-60.62
Lehouck et al. 2009 a	*Xymalos monospora*	dispersal	2.06	0.27	0.96	+	TRY	-3.33	38.25
Lehouck et al. 2009 a	*Xymalos monospora*	dispersal	0.61	0.18	0.96	+	TRY	-3.33	38.25
Lehouck et al. 2009 b	*Xymalos monospora*	recruitment	-0.3	0.51	0.96	+	TRY	-3.33	38.25
Leite et al. 2013	*Miconia albicans*	predation	-1.88	0.31	0.33	*	Christianini et al 2007	-22.83	-47.11
Lopes and Buzato 2007	*Psychotria suterella*	pollination	2.04	0.98	0.4	*		-23.73	-47.05
Mendes et al. 2015	*Syagrus romanzoffiana*	predation	0.26	0.86	2.50	+	TRY, daSilva et al. 2011	-22.66	-48.18
Menke et al. 2012	*Antiaris toxicaria*	dispersal	1.01	0.89	1.25	+	TRY	0.29	34.79
Menke et al. 2012	*Antiaris toxicaria*	dispersal	0.52	0.72	1.25	+	TRY	0.29	34.79
Menke et al. 2012	*Bridelia micrantha*	dispersal	0.66	0.76	0.86	+	TRY	0.29	34.79
Menke et al. 2012	*Bridelia micrantha*	dispersal	0.26	0.68	0.86	+	TRY	0.29	34.79
Menke et al. 2012	*Ficus lutea*	dispersal	-0.53	0.73	0.15			0.29	34.79
Menke et al. 2012	*Ficus lutea*	dispersal	0.82	0.81	0.15			0.29	34.79
Menke et al. 2012	*Ficus thonningii*	dispersal	2.79	2.34	0.12	+	TRY	0.29	34.79
Menke et al. 2012	*Ficus thonningii*	dispersal	1.16	0.95	0.12	+	TRY	0.29	34.79
Menke et al. 2012	*Harungana madagascariensis*	dispersal	2.78	2.32	0.28	+	TRY	0.29	34.79
Menke et al. 2012	*Harungana madagascariensis*	dispersal	1.35	1.06	0.28	+	TRY	0.29	34.79
Menke et al. 2012	*Polyscias fulva*	dispersal	0.54	0.73	0.36			0.29	34.79
Menke et al. 2012	*Polyscias fulva*	dispersal	-0.34	0.69	0.36			0.29	34.79
Menke et al. 2012	*Prunus africana*	dispersal	1.27	1.01	0.81	+	TRY	0.29	34.79
Menke et al. 2012	*Prunus africana*	dispersal	-0.04	0.67	0.81	+	TRY	0.29	34.79
Menke et al. 2012	*Trilepisium madagascariense*	dispersal	-0.5	0.72	1.57	+	TRY	0.29	34.79
Naniwadekaret al. 2015	*Phoebe sp.*	dispersal	-0.64	0.40	2.78			27.38	96.25
Naniwadekaret al. 2015	*Beilschmiedia assamica*	dispersal	-1.41	0.49	3.43			27.38	96.25
Naniwadekaret al. 2015	*Dysoxylum sp.*	dispersal	-0.78	0.41	2.83			27.38	96.25
Naniwadekaret al. 2015	*Canarium strictum*	dispersal	-0.57	0.39	3.38			27.38	96.25
Naniwadekaret al. 2015	*Alseodaphne petiolaris*	dispersal	-0.83	0.42	3.56			27.38	96.25

Naniwadekaret al. 2015	*Phoebe sp.*	recruitment	-4.09	1.39	2.78			27.38	96.25
Naniwadekaret al. 2015	*Beilschmiedia assamica*	recruitment	-12.57	9.93	3.43			27.38	96.25
Naniwadekaret al. 2015	*Dysoxylum sp.*	recruitment	-5.87	2.45	2.83			27.38	96.25
Naniwadekaret al. 2015	*Phoebe sp.*	recruitment	5.78	2.39	2.78			27.38	96.25
Naniwadekaret al. 2015	*Beilschmiedia assamica*	recruitment	-13.86	11.99	3.43			27.38	96.25
Naniwadekaret al. 2015	*Dysoxylum sp.*	recruitment	-1.91	0.59	2.83			27.38	96.25
Neuschulz et al. 2011	*Celtis africana*	dispersal	0.77	0.37	0.24	+	TRY, Markl et al. 2012	-30.47	30.44
Neuschulz et al. 2011	*Celtis africana*	dispersal	0.42	0.34	0.24	+	TRY, Markl et al. 2012	-30.47	30.44
Neuschulz et al. 2013	*Celtis africana*	pollination	0.37	0.34	0.24	+	TRY, Markl et al. 2012	-30.47	30.44
Neuschulz et al. 2013	*Celtis africana*	pollination	-0.66	0.4	0.24	+	TRY, Markl et al. 2012	-30.47	30.44
Nystrand and Granstroem 2000	*Pinus sylvestris*	predation	-0.66	0.15	0.36	*	TRY	63.98	19.78
O'Connell et al. 2006	*Picea glauca*	pollination	-1.23	0.07	0.22	*		46.33	-80.17
Quesada et al. 2003	*Ceiba aesculifolia*	pollination	-2.49	0.21	0.8	+	TRY, Markl et al. 2012	19.5	-105.05
Quesada et al. 2004	*Ceiba aesculifolia*	pollination	-0.34	0.16	0.8	+	TRY, Markl et al. 2012	12.89	-91.64
Quintana-Ascencio et al. 2004	*Abies guatemalensis*	recruitment	1.03	2.06	3		Andersen et al. 2006	16.73	-92.63
Quintana-Ascencio et al. 2004	*Myrsine juergensenii*	recruitment	1.19	2.42	1.02	*	Saldaña-Acosta et al. 2008	16.73	-92.63
Quintana-Ascencio et al. 2004	*Pinus ayacahuite*	recruitment	4.97	25.72	0.66	*	TRY	16.73	-92.63
Quintana-Ascencio et al. 2004	*Pinus pseudostrobus*	recruitment	1.93	4.73	0.47	*	TRY	16.73	-92.63
Quintana-Ascencio et al. 2004	*Ternstroemia lineata*	recruitment	0.42	1.18	1.48	*	Saldaña-Acosta et al. 2008	16.73	-92.63
Ramos and Santos 2006	*Psychotria tenuinervis*	pollination	-0.19	0.4	4.19	*	Ramos et al. 2007	-22.83	-42.47
Ramos and Santos 2006	*Psychotria tenuinervis*	pollination	-0.03	0.4	4.19	*	Ramos et al. 2007	-22.83	-42.47
Ratiarison and Forget 2005	*Tetragastris altissima*	dispersal	-0.45	0.47	1.6	+	TRY	4.85	-53.07
Ratiarison and Forget 2005	*Tetragastris altissima*	dispersal	-1.12	0.57	1.6	+	TRY	4.85	-53.07
Rivett et al. 2016	*Chlorocardium rodiei*	recruitment	0.47	0.10	6.31	*		4.67	-58.68
Rivett et al. 2016	*Eperua falcata*	recruitment	2.92	0.22	3.36	*		4.67	-58.68

Rivett et al. 2016	*Dicorynia guianensis*	recruitment	-0.67	0.11	1.82	*		4.67	-58.68
Rivett et al. 2016	*Goupia glabra*	recruitment	10.40	1.59	0.18	*		4.67	-58.68
Rodriguez-Oseguera et al. 2013	*Stenocereus quevedonis*	pollination	-4.3	0.41	0.2			19.07	-102.36
Roldan and Simonetti 2000	*Astrocaryum murumuru*	dispersal	-5.53	0.52	3.75	+	TRY	-14.67	-66.34
Roldan and Simonetti 2000	*Astrocaryum murumuru*	predation	-1.18	0.12	3.75	+	TRY	-14.67	-66.34
Saavedra 2015	*Clusia sp.*	recruitment	0.3	0.08	0.48	+	Personal comm.	-16.41	-67.53
Saavedra 2015	*Clusia sp.*	recruitment	0.76	0.09	0.48	+	Personal comm.	-16.41	-67.53
Santos and Telleria 1994	*Juniperus thurifera*	dispersal	-0.65	0.6	0.48	+	TRY	42.08	-3.75
Santos and Telleria 1994	*Juniperus thurifera*	predation	1.16	0.73	0.48	*	TRY	42.08	-3.75
Santos and Telleria 1994	*Juniperus thurifera*	recruitment	-0.8	0.68	0.48	+	TRY	42.08	-3.75
Santos and Telleria 1997	*Quercus ilex*	predation	0.59	0.45	2.96	+	TRY, Markl et al. 2012	42.02	-3.76
Santos and Telleria 1997	*Quercus ilex*	recruitment	0.91	0.48	2.96	+	TRY, Markl et al. 2012	42.02	-3.76
Santos and Telleria 1997	*Quercus ilex*	recruitment	-2.17	0.69	2.96	+	TRY, Markl et al. 2012	42.02	-3.76
Serio-Silva and Rico-Gray 2002	*Ficus americana*	dispersal	1.59	0.25	0.15			18.35	-95.06
Serio-Silva and Rico-Gray 2002	*Ficus aurea*	dispersal	0.44	0.23	0.15			18.35	-95.06
Sethi and Howe 2009	*Chisocheton cumingianus subsp. balansae*	dispersal	-0.45	0.1	3.1	+	TRY	27.09	92.86
Sethi and Howe 2009	*Chisocheton cumingianus subsp. balansae*	recruitment	-1.52	0.13	3.1	+	TRY	27.09	92.86
Sethi and Howe 2009	*Chisocheton cumingianus subsp. balansae*	recruitment	-1.03	0.11	3.1	+	TRY	27.09	92.86
Sethi and Howe 2009	*Dysoxylum gotadhora*	dispersal	-0.2	0.1	2.2	+	TRY	27.09	92.86
Sethi and Howe 2009	*Dysoxylum gotadhora*	recruitment	-1.41	0.13	2.2	+	TRY	27.09	92.86
Sethi and Howe 2009	*Dysoxylum gotadhora*	recruitment	-0.81	0.11	2.2	+	TRY	27.09	92.86
Sethi and Howe 2009	*Polyalthia simiarum*	dispersal	0.06	0.1	2.18	*	TRY	27.09	92.86
Sethi and Howe 2009	*Polyalthia simiarum*	recruitment	-1.88	0.15	2.18	*	TRY	27.09	92.86
Sethi and Howe 2009	*Polyalthia simiarum*	recruitment	-1.3	0.12	2.18	*	TRY	27.09	92.86
Shen et al. 2012	*Corylus heterophylla*	predation	-0.17	0.1	1.58	+		47.06	129.01

Shen et al. 2012	*Corylus mandshurica*	predation	0.03	0.1	1.43	+		47.06	129.01
Shen et al. 2012	*Juglans mandshurica*	predation	0.03	0.1	4.19	+		47.06	129.01
Shen et al. 2012	*Pinus koraiensis*	predation	-0.1	0.1	1.6	+		47.06	129.01
Shen et al. 2012	*Quercus mongolica*	predation	-0.34	0.1	2.52	+		47.06	129.01
Sica et al. 2014	*Syagrus romanzoffiana*	recruitment	-1.60	0.60	2.50	+	TRY, daSilva et al. 2011	-25.67	-54.40
Smith-Ramirez and Armesto 2003	*Embothrium coccineum*	pollination	0.72	0.18	0.4	*	TRY	-41.83	-73.58
Smith-Ramirez and Armesto 2003	*Embothrium coccineum*	pollination	-2.23	0.25	0.4	*	TRY	-41.83	-73.58
Sork et al. 1987	*Gustavia superba*	recruitment	0.9	0.05	2.93	*	TRY	9.12	-79.85
Suarez-Gonzalez and Good 2014	*Prunus virginiana*	pollination	-2.87	1.05	0.73	*	TRY	50.27	-96.9
Sugiyama and Peterson 2013	*Tapirira mexicana*	recruitment	-0.74	0.87	1.5	+	TRY	8.78	-82.97
Sugiyama and Peterson 2013	*Tapirira mexicana*	recruitment	-1.65	1.33	1.5	+	TRY	8.78	-82.97
Takeuchi at al. 2013	*Shorea laxa*	predation	-9.98	3.33	4.25	*	Tageuchi & Kashizuka 2007	4.03	113.83
Tokumoto et al. 2013	*Dillenia suffruticosa*	pollination	-1.46	0.2	0.33	*	TRY	4.25	113.98
Torella et al. 2015	*Aspidosperma quebracho-blanco*	recruitment	-0.52	0.15	6.00			-27.08	-61.00
Torella et al. 2015	*Caesalpinia paraguariensis*	recruitment	-0.96	0.16	1.00			-27.08	-61.00
Torella et al. 2015	*Prosopis kuntzei*	recruitment	-0.11	0.14	1.30			-27.08	-61.00
Torella et al. 2015	*Schinopsis lorentzii*	recruitment	-0.85	0.16	0.70			-27.08	-61.00
Torella et al. 2015	*Ziziphus mistol*	recruitment	-0.13	0.14	0.70			-27.08	-61.00
Torella et al. 2015	*Cordia americana*	recruitment	0.97	0.16	0.60			-27.08	-61.00
Torella et al. 2015	*Schinopsis balansae*	recruitment	-0.62	0.15	0.60			-27.08	-61.00
Traveset et al. 2003	*Rhamnus ludovici-salvatoris*	predation	0.59	0.09	0.25	*		39.82	2.88
Valdivia and Simonetti 2007	*Aristotelia chilensis*	dispersal	-1.45	0.16	0.2	+	TRY	-35.98	-72.68
Valdivia and Simonetti 2007	*Aristotelia chilensis*	recruitment	0.31	0.1	0.2	+	TRY	-35.98	-72.68
Valdivia et al. 2006	*Lapageria rosea*	pollination	-0.43	0.05	0.48	*	Henríquez 2004	-35.98	-72.68
Wang et al. 2005	*Ficus racemosa*	pollination	-0.18	0.45	0.15	*		21.68	101.42
Wang et al. 2007	*Antrocaryon klaineanum*	dispersal	-7.3	3.55	2.3			3.36	12.84
Wolowski and Freitas 2011	*Senna multijuga*	pollination	-0.58	0.36	0.32	*	TRY	-22.42	-44.57

Wolowski and Freitas 2011	*Senna multijuga*	pollination	-0.17	0.34	0.32	*	TRY	-22.42	-44.57
Wright and Duber 2001	*Attalea butyracea*	dispersal	-1.2	0.7	4	+	Wright et al. 2000	9.12	-79.71
Wright and Duber 2001	*Attalea butyracea*	predation	3.21	1.74	4	+	Wright et al. 2000	9.12	-79.71
Wright and Duber 2001	*Attalea butyracea*	predation	-5.08	3.54	4	+	Wright et al. 2000	9.12	-79.71
Wright and Duber 2001	*Attalea butyracea*	predation	-1.24	0.71	4	+	Wright et al. 2000	9.12	-79.71
Wright et al. 2000	*Astrocaryum standleyanum*	dispersal	-1.07	0.49	3	+	TRY	9.12	-79.71
Wright et al. 2000	*Astrocaryum standleyanum*	predation	0.91	0.29	3	+	TRY	9.12	-79.71
Wright et al. 2000	*Astrocaryum standleyanum*	predation	-1.49	0.33	3	+	TRY	9.12	-79.71
Wright et al. 2000	*Astrocaryum standleyanum*	recruitment	1.03	0.38	3	+	TRY	9.12	-79.71
Wright et al. 2000	*Attalea butyracea*	dispersal	-0.46	0.42	4	+	Wright et al. 2000	9.12	-79.71
Wright et al. 2000	*Attalea butyracea*	predation	0.58	0.28	4	+	Wright et al. 2000	9.12	-79.71
Wright et al. 2000	*Attalea butyracea*	predation	-2.19	0.41	4	+	Wright et al. 2000	9.12	-79.71
Wright et al. 2000	*Attalea butyracea*	recruitment	0.5	0.34	4	+	Wright et al. 2000	9.12	-79.71
Zambrano et al. 2015	*Poulsenia armata*	predation	-0.93	0.60	0.90	+	TRY	18.5	-95.07
Zambrano et al. 2015	*Poulsenia armata*	predation	-3.37	1.82	0.90	+	TRY	18.5	-95.07
Zambrano et al. 2014	*Poulsenia armata*	recruitment	-1.31	0.23	0.9	+	TRY	18.5	-95.07
Zambrano and Salguero-Gomez 2014	*Poulsenia armata*	recruitment	-0.22	0.51	0.90	+	TRY	18.5	-95.07

List of original publications included in the meta-analysis on the effects of human disturbance on plant regeneration processes.

Aguilar, R. & Galetto, L. Effects of forest fragmentation on male and female reproductive success in *Cestrum parqui* (Solanaceae). *Oecologia* **138,** 513–520 (2004).

Aguirre, A. & Dirzo, R. Effects of fragmentation on pollinator abundance and fruit set of an abundant understory palm in a Mexican tropical forest. *Biol. Conserv.* **141,** 375–384 (2008).

Aizen, M. A. & Feinsinger, P. Forest fragmentation, and plant reproduction in a Chaco dry forest, Argentina. *Ecology* **75,** 330–351 (1994).

Albrecht, J., Neuschulz, E. L. & Farwig, N. Impact of habitat structure and fruit abundance on avian seed dispersal and fruit predation. *Basic Appl. Ecol.* **13,** 347–354 (2012).

Aliaga-Rossel, E. & Fragoso, J. M. Defaunation affects *Astrocaryum gratum* (Arecales: Arecaceae) seed survivorship in a sub-montane tropical forest. *Int. J. Trop. Biol. Conserv.* **63,** 57–67 (2014).

Alvarez-Aquino, C., Williams-Linera, G. & Newton, A. C. Experimental native tree seedling establishment for the restoration of a Mexican cloud forest. *Restor. Ecol.* **12,** 412–418 (2004).

Andreazzi, C. S. *et al.* Increased productivity and reduced seed predation favor a large-seeded palm in small Atlantic forest fragments. *Biotropica* **44,** 237–245 (2012).

Anzures-Dadda, A., Andresen, E., Martínez, M. L. & Manson, R. H. Absence of howlers (*Alouatta palliata*) influences tree seedling densities in tropical rain forest fragments in Southern Mexico. *Int. J. Primatol.* **32,** 634–651 (2011).

Asbjornsen, H., Vogt, K. A. & Ashton, M. S. Synergistic responses of oak, pine and shrub seedlings to edge environments and drought in a fragmented tropical highland oak forest, Oaxaca, Mexico. *For. Ecol. Manage.* **192,** 313–334 (2004).

Ashworth, L., Calviño, A., Martí, M. L. & Aguilar, R. Offspring performance and recruitment of the pioneer tree Acacia caven (Fabaceae) in a fragmented subtropical dry forest. Austral Ecol. 40, 634–641 (2015).

Ashworth, L. & Martí, M. L. Forest fragmentation and seed germination of native species from the Chaco Serrano Forest. *Biotropica* **43,** 496–503 (2011).

Athayde, E. A. & Morellato, L. P. C. Anthropogenic edges, isolation and the flowering time and fruit set of *Anadenanthera peregrina*, a cerrado savanna tree. *Int. J. Biometeorol.* **58,** 443–54 (2014).

Avalos, G., Fernandez Otarola, M. & Engeln, J. T. Successional stage, fragmentation and exposure to extraction influence the population structure of *Euterpe precatoria* (Arecaeae). *Rev. Biol. Trop.* **61,** 1415–1424 (2013).

Babweteera, F. *Cordia millenii*: on the risk of local extinction? *Afr. J. Ecol.* **47,** 367–373 (2009).

Babweteera, F. & Brown, N. Can remnant frugivore species effectively disperse tree seeds in secondary tropical rain forests? *Biodivers. Conserv.* **18,** 1611–1627 (2009).

Bagchi, R. *et al.* Impacts of logging on density-dependent predation of dipterocarp seeds in a South East Asian rainforest. *Philos. Trans. R. Soc. B Biol. Sci.* **366,** 3246–3255 (2011).

Barbeta, A., Peñuelas, J., Ogaya, R. & Jump, A. S. Reduced tree health and seedling production in fragmented *Fagus sylvatica* forest patches in the Montseny Mountains (NE Spain). *For. Ecol. Manage.* **261,** 2029–2037 (2011).

Barrera Zambrano, V. A., Zambrano Moncada, J. & Stevenson, P. R. Diversity of regenerating plants and seed dispersal in two canopy trees from Colombian Amazon forests with different hunting pressure. *Rev. Biol. Trop.* **56,** 1531–1542 (2008).

Beckman, N. G. & Muller-Landau, H. C. Differential effects of hunting on pre dispersal seed predation and primary and secondary seed removal of two neotropical tree species. *Biotropica* **39,** 328–339 (2007).

Boissier, O., Bouiges, A., Mendoza, I., Feer, F. & Forget, P. M. Rapid assessment of seed removal and frugivore activity as a tool for monitoring the health status of tropical forests. Biotropica 46, 633–641 (2014).

Bose, A. K., Harvey, B. D. & Brais, S. Sapling recruitment and mortality dynamics following partial harvesting in aspen-dominated mixedwoods in eastern Canada. For. Ecol. Manage. 329, 37–48 (2014).

Braun, M. & Gottsberger, G. Reproduction of beetle-pollinated *Anaxagorea dolichocarpa* (Annonaceae) is resilient to habitat disturbance in rainforest fragments. *Nord. J. Bot.* **30,** 453–460 (2012).

Braun, M., Piechowski, D., Kazda, M. & Gottsberger, G. Fragment size and local flower density influence seed set of the understorey tree *Paypayrola blanchetiana* (Violaceae) in Brazilian Atlantic rain forest. *J. Trop. Ecol.* **28,** 353–359 (2012).

Broadhurst, L. M. & Young, A. G. Reproductive constraints for the long-term persistence of fragmented *Acacia dealbata* (Mimosaceae) populations in southeast Australia. *Biol. Conserv.* **133,** 512–526 (2006).

Brodie, J. F., Helmy, O. E., Brockelman, W. Y. & Maron, J. L. Bushmeat poaching reduces the seed dispersal and population growth rate of a mammal-dispersed tree. *Ecol. Appl.* **19,** 854–863 (2009).

Brum, H. D. *et al.* Rainforest fragmentation and the demography of the economically important palm *Oenocarpus bacaba* in central Amazonia. *Plant Ecol.* **199,** 209–215 (2008).

Bruna, E. M. Effects of forest fragmentation on *Heliconia acuminata* seedling recruitment in central Amazonia. *Oecologia* **132,** 235–243 (2002).

Camison, A. *et al.* Regeneration dynamics of *Quercus pyrenaica* Willd: In the Central System (Spain). *For. Ecol. Manage.* **343,** 42–52 (2015).

Campana Camargo, J. L., Kossman Ferraz, I. D. & Imakawa, A. M. Rehabilitation of degraded areas of Central Amazonia using direct sowing of forest tree seeds. *Restor. Ecol.* **10,** 636–644 (2002).

Cancio, I. *et al.* Habitat loss exacerbates regional extinction risk of the keystone semiarid shrub *Ziziphus lotus* through collapsing the seed dispersal service by foxes (*Vulpes vulpes*). *Biodivers. Conserv.* **25,** 693–709 (2016).

Chacoff, N. P., Morales, J. M. & Vaquera, M. P. Efectos de la Fragmentacion Sobre la Aborcion y Depredacion de Semillas en el Chaco Serrano. *Biotropica* **36,** 109–117 (2004).

Chapman, C. A., Chapman, L. J., Vulinec, K., Zanne, A. & Lawes, M. J. Fragmentation and alteration of seed dispersal processes: an initial evaluation of dung beetles, seed fate, and seedling diversity. *Biotropica* **35,** 382–393 (2003).

Chinchilla, F. A. Seed predation by mammals in forest fragments in Monteverde, Costa Rica. *Rev. Biol. Trop.* **57,** 865–877 (2009).

Christianini, A. V. & Oliveira, P. S. Edge effects decrease ant-derived benefits to seedlings in a neotropical savanna. *Arthropod. Plant. Interact.* **7,** 191–199 (2013).

Cordeiro, N. J., Ndangalasi, H. J., McEntee, J. P. & Howe, H. F. Disperser limitation and recruitment of an endemic African tree in a fragmented landscape. *Ecology* **90,** 1030–1041 (2009).

Cordeiro, N. J. & Howe, H. F. Forest fragmentation severs mutualism between seed dispersers and an endemic African tree. *Proc. Natl. Acad. Sci. U. S. A.* **100,** 14052–14056 (2003).

Cramer, J. M., Mesquita, R. C. G. & Bruce Williamson, G. Forest fragmentation differentially affects seed dispersal of large and small-seeded tropical trees. *Biol. Conserv.* **137,** 415–423 (2007a).

Cramer, J. M. *et al.* Forest fragmentation reduces seed dispersal of *Duckeodendron cestroides*, a Central Amazon endemic. *Biotropica* **39,** 709–718 (2007b).

Cunningham, S. A. Effects of habitat fragmentation on the reproductive ecology of four plant species in mallee woodland. *Conserv. Biol.* **14,** 758–768 (2000).

Da Silva, F. R., Begnini, R. M., Lopes, B. C. & Castellani, T. T. on two islands with different faunal richness, southern Brazil. *Stud. Neotrop. Fauna Environ.* **46,** 163–171 (2011).

Dausmann, K. H., Glos, J., Linsenmair, K. E. & Ganzhorn, J. U. Improved recruitment of a lemur-dispersed tree in Malagasy dry forests after the demise of vertebrates in forest fragments. *Oecologia* **157,** 307–316 (2008).

Del-Val, E., Armesto, J. J., Barbosa, O. & Marquet, P. A. Effects of herbivory and patch size on tree seedling survivorship in a fog-dependent coastal rainforest in semiarid Chile. *Oecologia* **153,** 625–632 (2007).

Donatti, C. I., Guimaraes, J. P. R. & Galetti, M. Seed dispersal and predation in the endemic Atlantic rainforest palm *Astrocaryum aculeatissimum* across a gradient of seed disperser abundance. *Ecol. Res.* **24,** 1187–1195 (2009).

Donoso, D. S., Grez, A. A. & Simonetti, J. A. Effects of forest fragmentation on the granivory of differently sized seeds. *Biol. Conserv.* **115,** 63–70 (2003).

Dunley, B. S., Freitas, L. & Galetto, L. Reproduction of *Byrsonima sericea* (Malpighiaceae) in Restinga fragmented habitats in Southeastern Brazil. *Biotropica* **41,** 692–699 (2009).

Fadini, R. F., Fleury, M., Donatti, C. I. & Galetti, M. Effects of frugivore impoverishment and seed predators on the recruitment of a keystone palm. *Acta Oecologica* **35,** 188–196 (2009).

Farwig, N., Bleher, B., Von Der Gönna, S. & Böhning-Gaese, K. Does forest fragmentation and selective logging affect seed predators and seed predation rates of *Prunus africana* (Rosaceae)? *Biotropica* **40,** 218–224 (2008).

Farwig, N., Böhning-Gaese, K. & Bleher, B. Enhanced seed dispersal of *Prunus africana* in fragmented and disturbed forests? *Oecologia* **147,** 238–252 (2006).

Figueroa-Esquivel, E., Puebla-Olivares, F., Godínez-Álvarez, H. & Núñez-Farfán, J. Seed dispersal effectiveness by understory birds on *Dendropanax arboreus* in a fragmented landscape. *Biodivers. Conserv.* **18,** 3357–3365 (2009).

Fleury, M. & Galetti, M. Forest fragment size and microhabitat effects on palm seed predation. *Biol. Conserv.* **131,** 1–13 (2006).

Fleury, M. & Galetti, M. Effects of microhabitat on palm seed predation in two forest fragments in southeast Brazil. *Acta Oecologica* **26,** 179–184 (2004).

Forget, P. M. & Jansen, P. A. Hunting increases dispersal limitation in the tree *Carapa procera*, a nontimber forest product. *Conserv. Biol.* **21,** 106–113 (2007).

Forget, P.-M. & Cuijpers, L. Survival and scatterhoarding of frugivores-dispersed seeds as a function of forest disturbance. *Biotropica* **40,** 380–385 (2008).

Forget, P.-M., Rankin-De Merona, J. M. & Juillot, C. The effects of forest type, harvesting and stand refinement on early seedling recruitment in a tropical rain forest. *J. Trop. Ecol.* **17,** (2001).

Franceschinelli, E. V. *et al.* Reproductive success of *Cabralea canjerana* (Meliaceae) in Atlantic forest fragments, Brazil. *Rev. Biol. Trop. (Int. J. Trop. Biol* **63,** 515–524 (2015).

Galetti, M., Bovendorp, R. S. & Guevara, R. Defaunation of large mammals leads to an increase in seed predation in the Atlantic forests. *Glob. Ecol. Conserv.* **3,** 824–830 (2015).

Galetti, M., Donatti, C. I., Pires, A. S., Guimarães, P. R. & Jordano, P. Seed survival and dispersal of an endemic Atlantic forest palm: The combined effects of defaunation and forest fragmentation. *Bot. J. Linn. Soc.* **151,** 141–149 (2006).

Gallegos, S. C., Hensen, I., Saavedra, F. & Schleuning, M. Bracken fern facilitates tree seedling recruitment in tropical fire-degraded habitats. *For. Ecol. Manage.* **337,** 135–143 (2015).

Gallegos, S. C., Hensen, I. & Schleuning, M. Secondary dispersal by ants promotes forest regeneration after deforestation. *J. Ecol.* **102,** 659–666 (2014).

Gallegos Ayala, S. C. Factors limiting forest regeneration in bracken dominated areas in the tropical montane forest of Bolivia. Doctoral thesis, University of Halle (2014).

Ghazoul, J., Liston, K. A. & Boyle, T. J. B. Disturbance-induced density-dependent seed set in *Shorea siamensis* (Dipterocarpaceae), a tropical forest tree. *J. Ecol.* **86,** 462–773 (1998).

Ghazoul, J. & McLeish, M. Reproductive ecology of tropical forest trees in logged and fragmented habitats in Thailand. *Plant Ecol.* **153,** 335–345 (2001).

González-Di Pierro, A. M. *et al.* Effects of the physical environment and primate gut passage on the early establishment of *Ampelocera hottlei standley* in rain forest fragments. *Biotropica* **43,** 459–466 (2011).

González-Varo, J. P. Fragmentation, habitat composition and the dispersal/predation balance in interactions between the mediterranean myrtle and avian frugivores. *Ecography.* **33,** 185–197 (2010).

González-Varo, J. P., Nora, S. & Aparicio, A. Bottlenecks for plant recruitment in woodland remnants: An ornithochorous shrub in a Mediterranean 'relictual' landscape. *Perspect. Plant Ecol. Evol. Syst.* **14,** 111–122 (2012).

Graham, C. H., Martínez-Leyva, J. E. & Cruz-Paredes, L. Use of fruiting trees by birds in continuous forest and riparian forest remnants in Los Tuxtlas, Veracruz, Mexico. *Biotropica* **34,** 589–597 (2002).

Guariguata, M. R., Arias-Le Claire, H. & Jones, G. Tree seed fate in a logged and fragmented forest landscape, northeastern Costa Rica. *Biotropica* **34,** 405–415 (2002).

Guariguata, M. R., Adame, J. J. R. & Finegan, B. Seed removal and fate in two selectively logged lowland forests with constrasting protection levels. *Conserv. Biol.* **14,** 1046–1054 (2000).

Guerrero, P. C. & Bustamante, R. O. Abiotic alterations caused by forest fragmentation affect tree regeneration: a shade and drought tolerance gradient in the remnants of Coastal Maulino Forest. *Rev. Chil. Hist. Nat.* **82,** 413–424 (2009).

Gutiérrez-Granados, G. & Dirzo, R. Indirect effects of timber extraction on plant recruitment and diversity via reductions in abundance of frugivorous spider monkeys. *J. Trop. Ecol.* **26,** 45 (2010).

Gutiérrez-Granados, G., Juárez, V. & Alcalá, R. E. Natural and human disturbances affect natural regeneration of Swietenia macrophylla: Implications for rainforest management. *For. Ecol. Manage.* **262,** 161–169 (2011).

Hanson, T., Brunsfeld, S. & Finegan, B. Variation in seedling density and seed predation indicators for the emergent tree *Dipteryx panamensis* in continuous and fragmented rain forest. *Biotropica* **38,** 770–774 (2006).

Herrerías-Diego, Y., Quesada, M., Stoner, K. E. & Lobo, J. A. Effects of forest fragmentation on phenological patterns and reproductive success of the tropical dry forest tree *Ceiba aesculifolia*. *Conserv. Biol.* **20,** 1111–1120 (2006).

Hoebee, S. E. *et al.* Mating patterns and contemporary gene flow by pollen in a large continuous and a small isolated population of the scattered forest tree *Sorbus torminalis*. *Heredity.* **99,** 47–55 (2007).

Holbrook, K. M. & Loiselle, B. A. Dispersal in a Neotropical tree, *Virola flexuosa* (Myristicaceae): Does hunting of large vertebrates limit seed removal? *Ecology* **90,** 1449–1455 (2009).

Holl, K. D. & Lulow, M. E. Effects of species, habitat, and distance from edge on post-dispersal seed predation in a tropical rainforest. *Biotropica* **29,** 459–468 (1997).

Iob, G. & Vieira, E. M. Seed predation of *Araucaria angustifolia* (Araucariaceae) in the Brazilian Araucaria Forest: Influence of deposition site and comparative role of small and 'large' mammals. *Plant Ecol.* **198,** 185–196 (2008).

Ismail, S. A. *et al.* Forest trees in human modified landscapes: Ecological and genetic drivers of recruitment failure in *Dysoxylum malabaricum* (Meliaceae). *PLoS One* **9,** (2014).

Jorge, M. L. S. P. & Howe, H. F. Can forest fragmentation disrupt a conditional mutualism? A case from central Amazon. *Oecologia* **161,** 709–718 (2009).

Khan, M. L., Bhuyan, P. & Tripathi, R. S. Effects of forest disturbance on fruit set, seed dispersal and predation of Rudraksh (*Elaeocarpus granitrus* Roxb.) in northeast India. *Curr. Sci.* **88,** 133–142 (2005).

Kirika, J. M., Bleher, B., Böhning-Gaese, K., Chira, R. & Farwig, N. Fragmentation and local disturbance of forests reduce frugivore diversity and fruit removal in *Ficus thonningii* trees. *Basic Appl. Ecol.* **9,** 663–672 (2008).

Lefevre, K. L. & Rodd, F. H. How human disturbance of tropical rainforest can influence avian fruit removal. *Oikos* **118,** 1405–1415 (2009).

Lehouck, V. *et al.* Habitat disturbance reduces seed dispersal of a forest interior tree in a fragmented African cloud forest. *Oikos* **118,** 1023–1034 (2009).

Lehouck, V., Spanhove, T., Gonsamo, A., Cordeiro, N. & Lens, L. Spatial and temporal effects on recruitment of an Afromontane forest tree in a threatened fragmented ecosystem. *Biol. Conserv.* **142,** 518–528 (2009).

Leite, R. R., Soares Cardoso de Araujo, S. & Gama de Oliveira, E. Remocao dos frutos de *Miconia albicans* (SW.) Triana (Melastomataceae) por formigas na borda e no interior de um fragmento de cerrado, curvelo, MG. *Rev. Árvore* **37,** 469–478 (2013).

Lopes, L. E. & Buzato, S. Variation in pollinator assemblages in a fragmented landscape and its effects on reproductive stages of a self-incompatible treelet, *Psychotria suterella* (Rubiaceae). *Oecologia* **154,** 305–314 (2007).

Mendes, C. P., Ribeiro, M. C. & Galetti, M. Patch size, shape and edge distance influence seed predation on a palm species in the Atlantic forest. *Ecography (Cop.).* **39,** 465–475 (2016).

Menke, S., Böhning-Gaese, K. & Schleuning, M. Plant-frugivore networks are less specialized and more robust at forest-farmland edges than in the interior of a tropical forest. *Oikos* **121,** 1553–1566 (2012).

Naniwadekar, R., Shukla, U., Isvaran, K. & Datta, A. Reduced hornbill abundance associated with low seed arrival and altered recruitment in a hunted and logged tropical forest. *PLoS One* **10,** 1–17 (2015).

Neuschulz, E. L., Grass, I., Botzat, A., Johnson, S. D. & Farwig, N. Persistence of flower visitors and pollination services of a generalist tree in modified forests. *Austral Ecol.* **38,** 374–382 (2013).

Neuschulz, E. L., Botzat, A. & Farwig, N. Effects of forest modification on bird community composition and seed removal in a heterogeneous landscape in South Africa. *Oikos* **120,** 1371–1379 (2011).

Nystrand, O. & Granström, A. Predation on *Pinus sylvestris* seeds and juvenile seedlings in Swedish boreal forest in relation to stand disturbance by logging. *J. Appl. Ecol.* **37,** 449–463 (2000).

O'Connell, L. M., Mosseler, A. & Rajora, O. P. Impacts of forest fragmentation on the reproductive success of white spruce (*Picea glauca*). *Can. J. Bot. Can. Bot.* **84,** 956–965 (2006).

Quesada, M. *et al.* Effects of forest fragmentation on pollinator activity and consequences for plant reproductive success and mating patterns in bat-pollinated bombacaceous trees. *Biotropica* **36,** 131–138 (2004).

Quesada, M., Stoner, K. E., Rosas-Guerrero, V., Palacios-Guevara, C. & Lobo, J. a. Effects of habitat disruption on the activity of nectarivorous bats (Chiroptera: Phyllostomidae) in a dry tropical forest: implications for the reproductive success of the neotropical tree *Ceiba grandiflora*. *Oecologia* **135,** 400–406 (2003).

Quintana-Ascencio, P. F., Ramírez-Marcial, N., González-Espinosa, M. & Martínez-Icó, M. Sapling survival and growth of coniferous and broad-leaved trees in successional highland habitats in Mexico. *Appl. Veg. Sci.* **7,** 81–88 (2004).

Ramos, F. N. & Santos, F. A. M. Floral visitors and pollination of *Psychotria tenuinervis* (Rubiaceae): Distance from the anthropogenic and natural edges of an Atlantic forest fragment. *Biotropica* **38,** 383–389 (2006).

Ratiarison, S. & Forget, P.-M. Frugivores and seed removal at *Tetragastris altissima* (Burseraceae) in a fragmented forested landscape of French Guiana. *J. Trop. Ecol.* **21,** 501–508 (2005).

Rivett, S. L., Bicknell, J. E. & Davies, Z. G. Effect of reduced-impact logging on seedling recruitment in a neotropical forest. *For. Ecol. Manage.* **367,** 71–79 (2016).

Rodríguez-Oseguera, A. G., Casas, A., Herrerías-Diego, Y. & Pérez-Negrón, E. Effect of habitat disturbance on pollination biology of the columnar cactus *Stenocereus quevedonis* at landscape-level in central Mexico. *Plant Biol.* **15,** 573–582 (2013).

Roldán, A. I. & Simonetti, J. A. Plant-mammal interactions in tropical Bolivian forests with different hunting pressures. *Conserv. Biol.* **15,** 617–623 (2001).

Saavedra, F. V. Seed dispersal by birds in tropical montane forests: towards a functional understanding of seed-dispersal effectiveness after deforestation. Doctoral thesis, University of Halle (2015).

Santos, T. & Tellería, J. L. Influence of forest fragmentation on seed consumption and dispersal of Spanish juniper *Juniperus thurifera*. *Biol. Conserv.* **70,** 129–134 (1994).

Santos, T. & Tellería, J. L. Vertebrate predation on holm oak, *Quercus ilex*, acorns in a fragmented habitat: Effects on seedling recruitment. *For. Ecol. Manage.* **1998,** 181–187 (1997).

Serio-Silva, J. C. & Rico-Gray, V. Interacting effects of forest fragmentation and howler monkey foraging on germination and disperal of fig seeds. *Oryx* **36,** 266–271 (2002).

Sethi, P. & Howe, H. F. Recruitment of hornbill-dispersed trees in hunted and logged forests of the Indian Eastern Himalaya. *Conserv. Biol.* **23,** 710–718 (2009).

Shen, Z., Guo, S., Yang, Y. & Yi, X. Decrease of large-bodied dispersers limits recruitment of large-seeded trees but benefits small-seeded trees. *Isr. J. Ecol. Evol.* **58,** 53–67 (2012).

Sica, Y. V., Bravo, S. P. & Giombini, M. I. Spatial pattern of Pindo palm (*Syagrus romanzoffiana*) recruitment in Argentinian atlantic forest: The importance of tapir and effects of defaunation. *Biotropica* **46,** 696–703 (2014).

Smith-Ramirez, C. & Armesto, J. J. Foraging behavior of bird pollinators on *Embothrium coccineum* (Proteaceae) trees in forest fragments and pastures in southern Chile. *Austral Ecol.* **28,** 53–60 (2003).

Sork, V. L. Effects of predation and light on seedling establishment in *Gustavia superba*. *Ecology* **68,** 1341–1350 (1987).

Suarez-Gonzalez, A. & Good, S. V. Pollen limitation and reduced reproductive success are associated with local genetic effects in *Prunus virginiana*, a widely distributed self-incompatible shrub. *Ann. Bot.* **113,** 595–605 (2014).

Sugiyama, A. & Peterson, C. J. Edge effects act differentially on multiple early regeneration stages of a shade-tolerant tree *Tapirira mexicana*. *Biotropica* **45,** 37–44 (2013).

Takeuchi, Y., Nakagawa, M., Diway, B. & Nakashizuka, T. Reproductive success of a tropical tree, *Shorea laxa*, in a pulau (forest reserve) managed by a local community in Borneo. *For. Ecol. Manage.* **289,** 416–424 (2013).

Tokumoto, Y., Itioka, T., Ohkubo, T., Tadauchi, O. & Nakagawa, M. Assemblage of flower visitors to *Dillenia suffruticosa* and possible negative effects of disturbances in Sarawak, Malaysia. *Entomol. Sci.* **16,** 341–351 (2013).

Torrella, S. A., Ginzburg, R. & Galetto, L. Forest fragmentation in the Argentine Chaco: recruitment and population patterns of dominant tree species. *Plant Ecol.* **216,** 1499–1510 (2015).

Traveset, A., Gulias, J., Riera, N. & Mus, M. Transition from pollination to establishment probabilities in a rare dioecious shrub species (*Rhamnus ludovici-salvatoris*) in two habitats. *J. Ecol.* **91,** 427–437 (2003).

Valdivia, C. E. & Simonetti, J. A. Decreased frugivory and seed germination rate do not reduce seedling recruitment rates of *Aristotelia chilensis* in a fragmented forest. *Biodivers. Conserv.* **16,** 1593–1602 (2007).

Valdivia, C. E., Simonetti, J. A. & Henríquez, C. A. Depressed pollination of *Lapageria rosea* Ruiz et Pav. (Philesiaceae) in the fragmented temperate rainforest of southern South America. *Biodivers. Conserv.* **15,** 1845–1856 (2006).

Wang, B. C., Sork, V. L., Leong, M. T. & Smith, T. B. Hunting of mammals reduces seed removal and dispersal of the afrotropical tree *Antrocaryon klaineanum* (Anacardiaceae). *Biotropica* **39,** 340–347 (2007).

Wang, R., Yang, C., Zhao, G. & Yang, J. Fragmentation effects on diversity of wasp community and its impact on fig/fig wasp interaction in *Ficus racemosa* L. *J. Integr. Plant Biol.* **47,** 20–26 (2005).

Wolowski, M. & Freitas, L. Reproduction, pollination and seed predation of *Senna multijuga* (Fabaceae) in two protected areas in the Brazilian Atlantic forest. *Rev. Biol. Trop.* **59,** 1939–1948 (2011).

Wright, S. J. & Duber, H. C. Poachers and forest fragmentation alter seed dispersal, seed survival, and seedling recruitment in the palm *Attalea butyraceae*, with implications for tropical tree diversity. *Biotropica* **33,** 583–595 (2001).

Wright, S. J. *et al.* Poachers alter mammal anundance, seed dispersal, and seed predation in a Neotropical forest. *Conserv. Biol.* **14,** 227–239 (2000).

Zambrano, J., Coates, R. & Howe, H. F. Seed predation in a human-modified tropical landscape. *J. Trop. Ecol.* **31,** 379–383 (2015).

Zambrano, J., Coates, R. & Howe, H. F. Effects of forest fragmentation on the recruitment success of the tropical tree *Poulsenia armata* at Los Tuxtlas, Veracruz, Mexico. *J. Trop. Ecol.* **30,** 209–218 (2014).

Zambrano, J. & Salguero-Gómez, R. Forest fragmentation alters the population dynamics of a late-successional tropical tree. *Biotropica* **46,** 556–564 (2014).

Databases and source publications of seed size / mass data used, if data on seed size / mass were not specified in the original publication

Databases:

TRY Kattge, J. *et al.* TRY - a global database of plant traits. *Glob. Chang. Biol.* **17,** 2905–2935 (2011).

Encyclopedia of life. Encyclopedia of Life. Available from http://www.eol.org. Accessed Nov 2014.

Source publications

Aliaga-Rossel, E. Phenology and germination of the Chonta palm *Astrocaryum gratum*, in a sub-montane forest. *Palms* **55,** 84–92 (2011).

Funes, G., Díaz, S. & Venier, P. La temperatura como principal determinante de la germinación en especies del Chaco seco de Argentina. *Ecol. Austral* **19,** 129–138 (2009).

Galetti, M. *et al.* The role of seed mass on the caching decision by agoutis, *Dasyprocta leporina* (Rodentia: Agoutidae). *Zool. (Curitiba, Impresso)* **27,** 472–476 (2010).

Green, D. F. & Johnson, E. A. Estimating the mean annual seed production of trees. *Ecology* **75,** 642–647 (1994).

Henríquez, C. A. Efecto de la fragmentación del hábitat sobre la calidad de las semillas en *Lapageria rosea*. *Rev. Chil. Hist. Nat.* **77,** 177–184 (2004).

Homeier, J., Breckle, S.-W., Dalitz, H., Leyers, C. & Ortiz, R. Demography, spatial distribution, and growth of three arborescent palm species in a tropical premontane rain forest in Costa Rica. *Ecotropica* **8,** 239–247 (2002).

Markl, J. S. *et al.* Meta-analysis of the effects of human disturbance on seed dispersal by animals. *Conserv. Biol.* **26,** 1072–81 (2012).

Marod, D., Kutintara, U., Tanaka, H. & Nakashiizuka, T. The effects of drought and fire on seed and seedling dynamics in a tropical seasonal forest in Thailand. *Plant Ecol.* **161,** 41–57 (2002).

O'Farrill, G., Chapman, C. A. & Gonzalez, A. Origin and deposition sites influence seed germination and seedling survival of *Manilkara zapota*: implications for long-distance, animal-mediated seed dispersal. *Seed Sci. Res.* **21,** 305–313 (2011).

Oyelade, O. J., Odugbenro, P. O., Abioye, A. O. & Raji, N. L. Some physical properties of African star apple (*Chrysophyllum alibidum*) seeds. *J. Food Eng.* **67,** 435–440 (2005).

Quero, L. Q., Villar, R., Marañón, T., Zamora, R. & Poorter, L. Seed-mass effects in four Mediterranean *Quercus* species (Fagaceae) growing in constrasting light environments. *Am. J. Bot.* **94,** 1795–1803 (2007).

Ramos, F. N. *et al.* Quality of seeds produced by *Psychotria tenuinervis* (Rubiaceae): distance from anthropogenic and natural edges of Atlantic forest fragment. *Biochem. Genet.* **45,** 441–458 (2007).

Reyes-Agüero, J. A., Aguirre R., J. R. & Valiente-Banuet, A. Reproductive biology of Opuntia: A review. *J. Arid Environ.* **64,** 549–585 (2006).

Saldaña-Acosta, A. *et al.* Variation of functional traits in trees from a biogeographically complex Mexican cloud forest. *Acta Oecologica* **34,** 111–121 (2008).

St-Denis, A., Messier, C. & Kneeshaw, D. Seed size, the only factor positively affecting direct seeding success in an abandoned field in Quebec, Canada. *Forests* **4,** 500–516 (2013).

Takeuchi, Y. & Nakasizuka, T. Effect of distance and density on seed/seedling fate of two dipterocarp species. *For. Ecol. Manage.* **247,** 167–174 (2007).
